# Supplementary material for: Deep eutectic solvent for an expeditious sono-synthesis of novel series of bis-quinazolin-4-one derivatives as potential anti-cancer agents
Source: R Soc Open Sci. 2019 Mar 20;6(3):182046. doi: 10.1098/rsos.182046 (PMC6458391; doi:10.1098/rsos.182046)
Supplement: Supplementary material [file rsos182046supp1.pdf]

Electronic Supporting Information

Deep eutectic solvent for an expeditious sono-synthesis of novel series of bis-quinazolin-4-one derivatives as potential anticancer agents

Wael Abdelgayed Ahmed Arafa

Chemistry Department, College of Science, Jouf University, P.O. Box 2014, Sakaka, Aljouf, Kingdom of Saudi Arabia

Chemistry Department, Faculty of Science, Fayoum University P.O. Box 63514, Fayoum City, Egypt

E-mail: waa00@fayoum.edu.eg

Index

No.	Content	Page No.
1.	Experimental details for the synthesis of **1a-e**	S1
2.	Copies of ^{1}H NMR and ^{13}C NMR of the synthesized products	S2-S58
3.	**Table 3.** The anti-proliferative activities of the newly prepared bis-quinazolinone derivatives against human cancer cell lines.	S59
4.	Green metrics calculations	S62
5.	References	S62

Synthesis of 1a-e

For the synthesis of derivatives **1a-e**, a mixture of anthranilic acid derivatives (1 mmol) and acetic anhydride (≈ 10 mL, 10 mmol) was sonicated at 40 °C for 10 min. The excess of acetic anhydride was distilled off under vacuum and the residue was dissolved in DCM. The obtained crystals filtered, dried and used for the next steps.

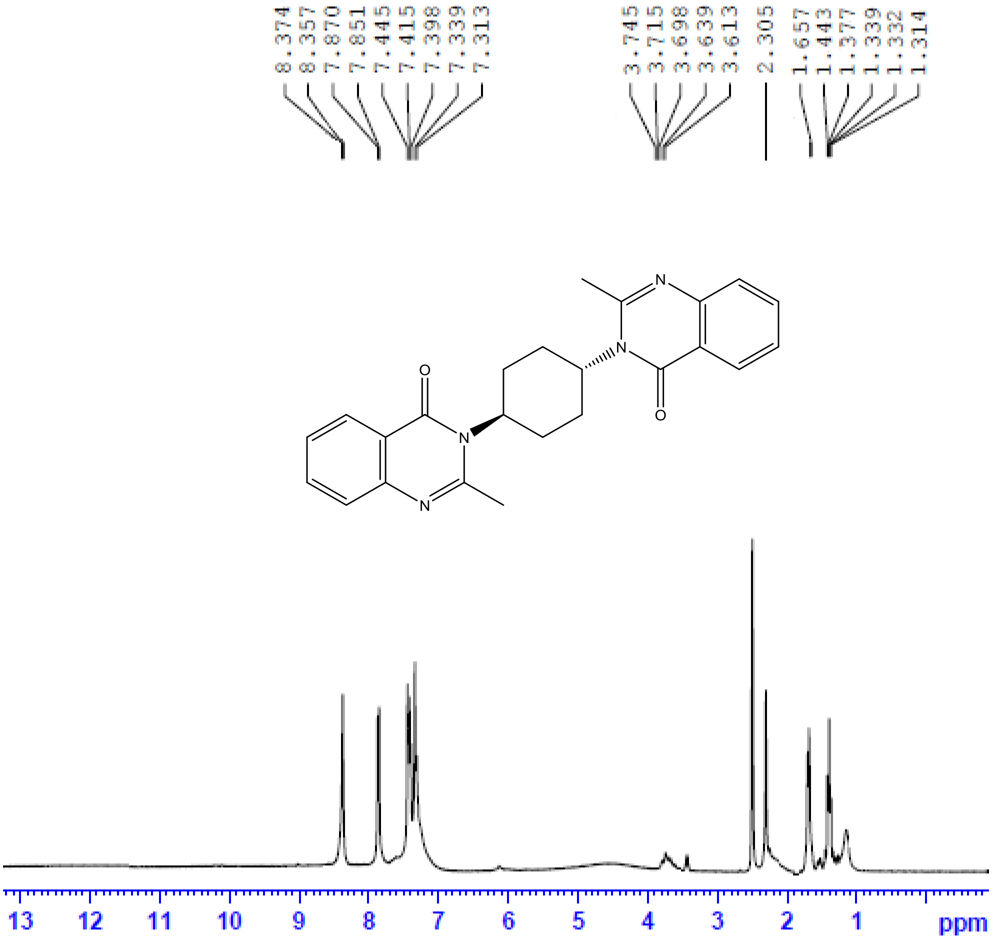
8.374
8.357
7.870
7.851
7.445
7.415
7.398
7.339
7.313
3.745
3.715
3.698
3.639
3.613
2.305
1.657
1.443
1.377
1.339
1.332
1.314
N
N
O
N
N
O
13 12 11 10 9 8 7 6 5 4 3 2 1 ppm

^{1}H NMR of **3a**

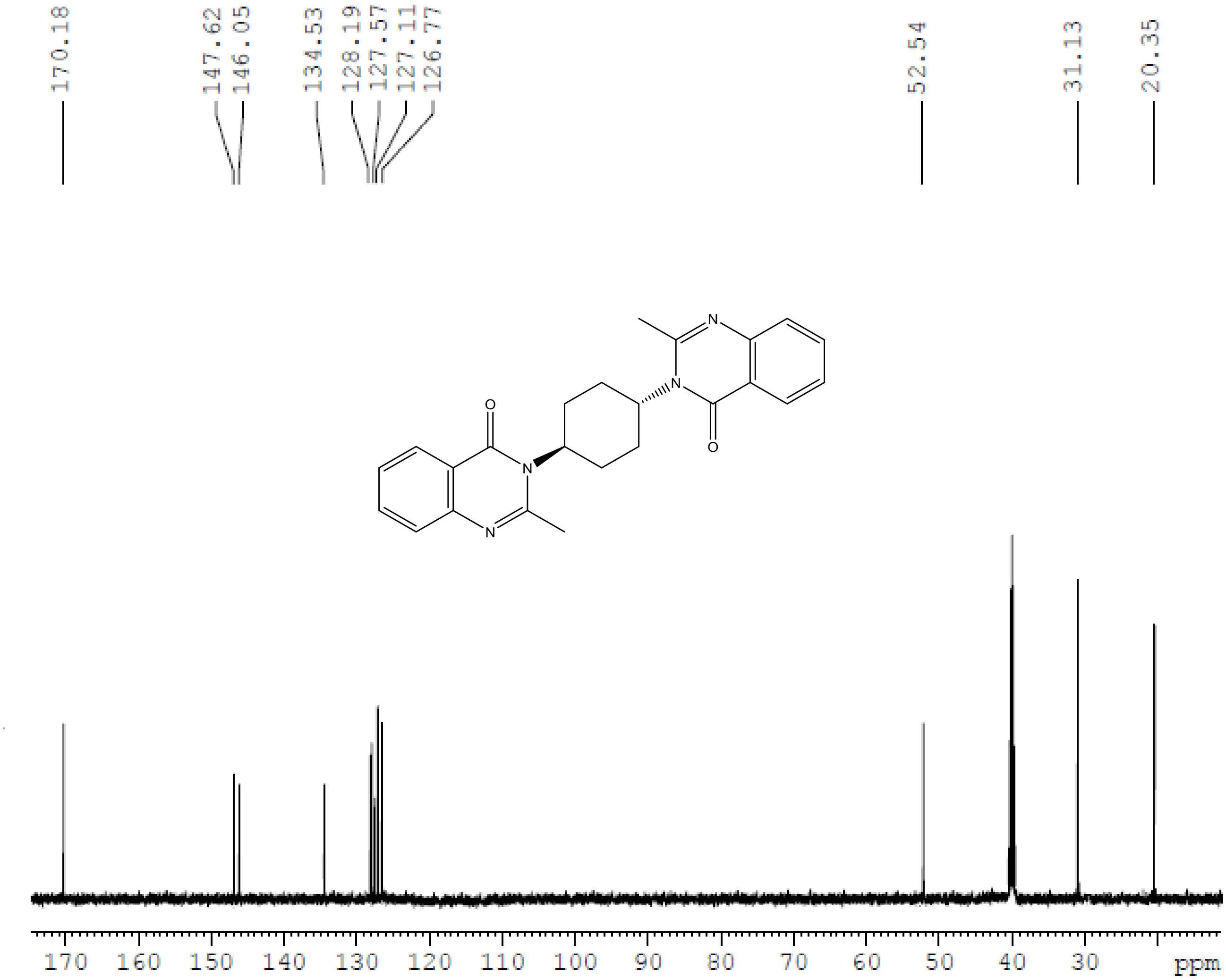
170.18
147.62
146.05
134.53
128.19
127.57
127.11
126.77
52.54
31.13
20.35
O
N
N
N
N
O
170
160
150
140
130
120
110
100
90
80
70
60
50
40
30
ppm

^{13}C NMR of **3a**

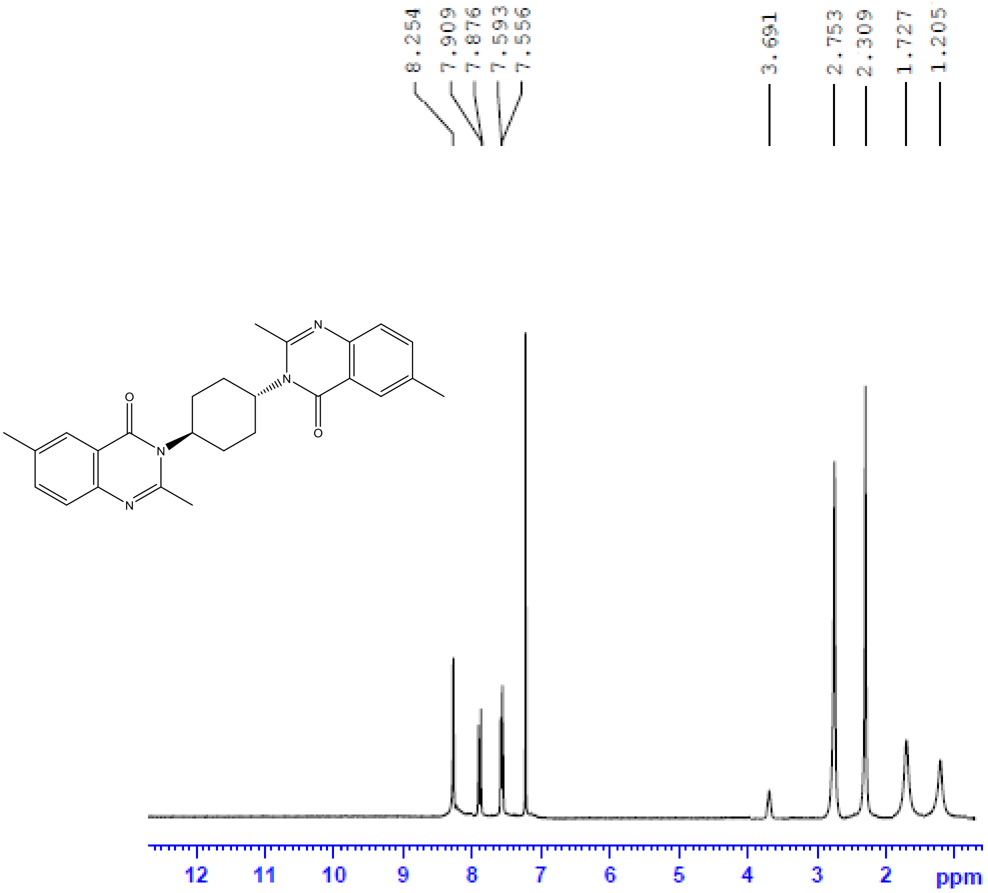
8.254
7.909
7.876
7.593
7.556
3.691
2.753
2.309
1.727
1.205
12
11
10
9
8
7
6
5
4
3
2
ppm

^{1}H NMR of **3b**

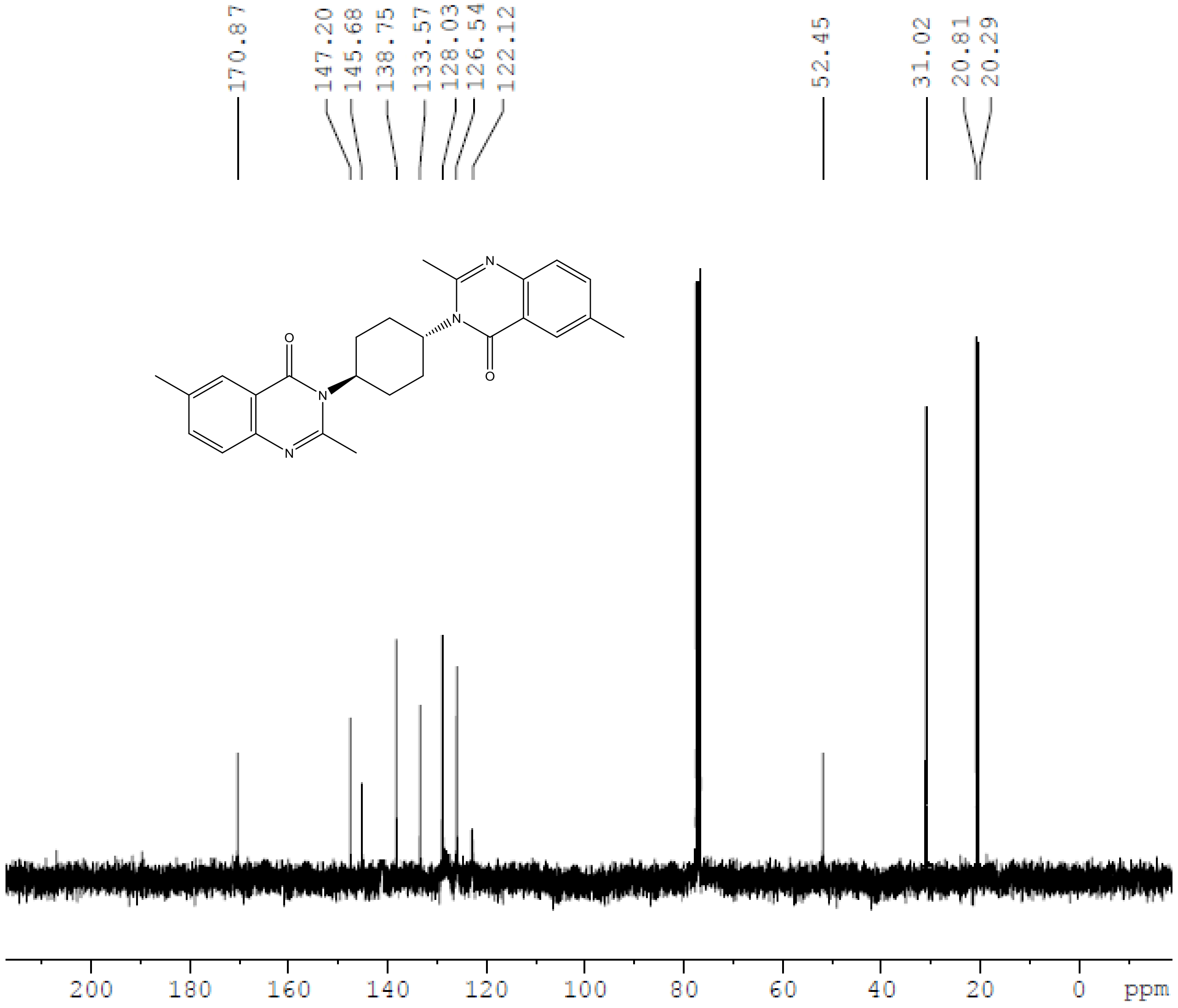
170.87
147.20
145.68
138.75
133.57
128.03
126.54
122.12
52.45
31.02
20.81
20.29
O
N
N
N
N
O
200
180
160
140
120
100
80
60
40
20
0
ppm

^{13}C NMR of **3b**

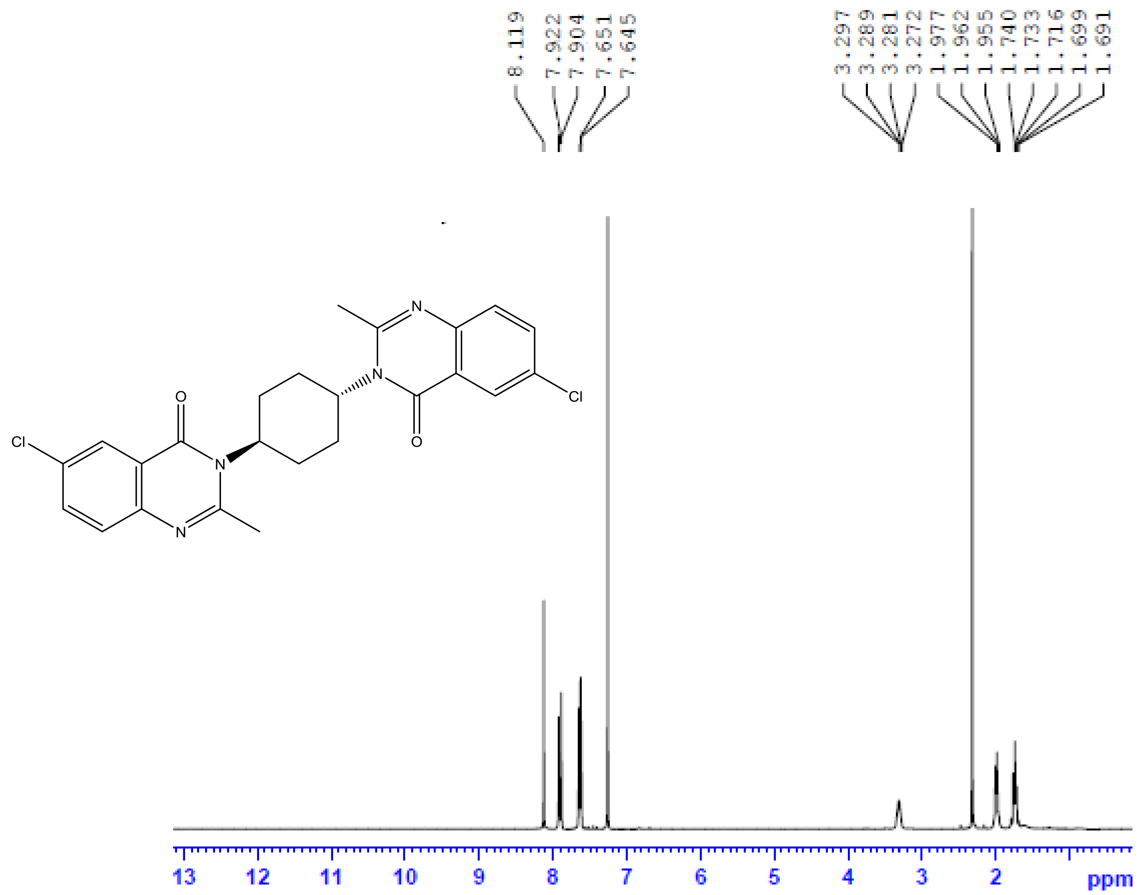
8.119
7.922
7.904
7.651
7.645
3.297
3.289
3.281
3.272
1.977
1.962
1.955
1.740
1.733
1.716
1.699
1.691
Cl
N
O
ppm

^{1}H NMR of **3c**

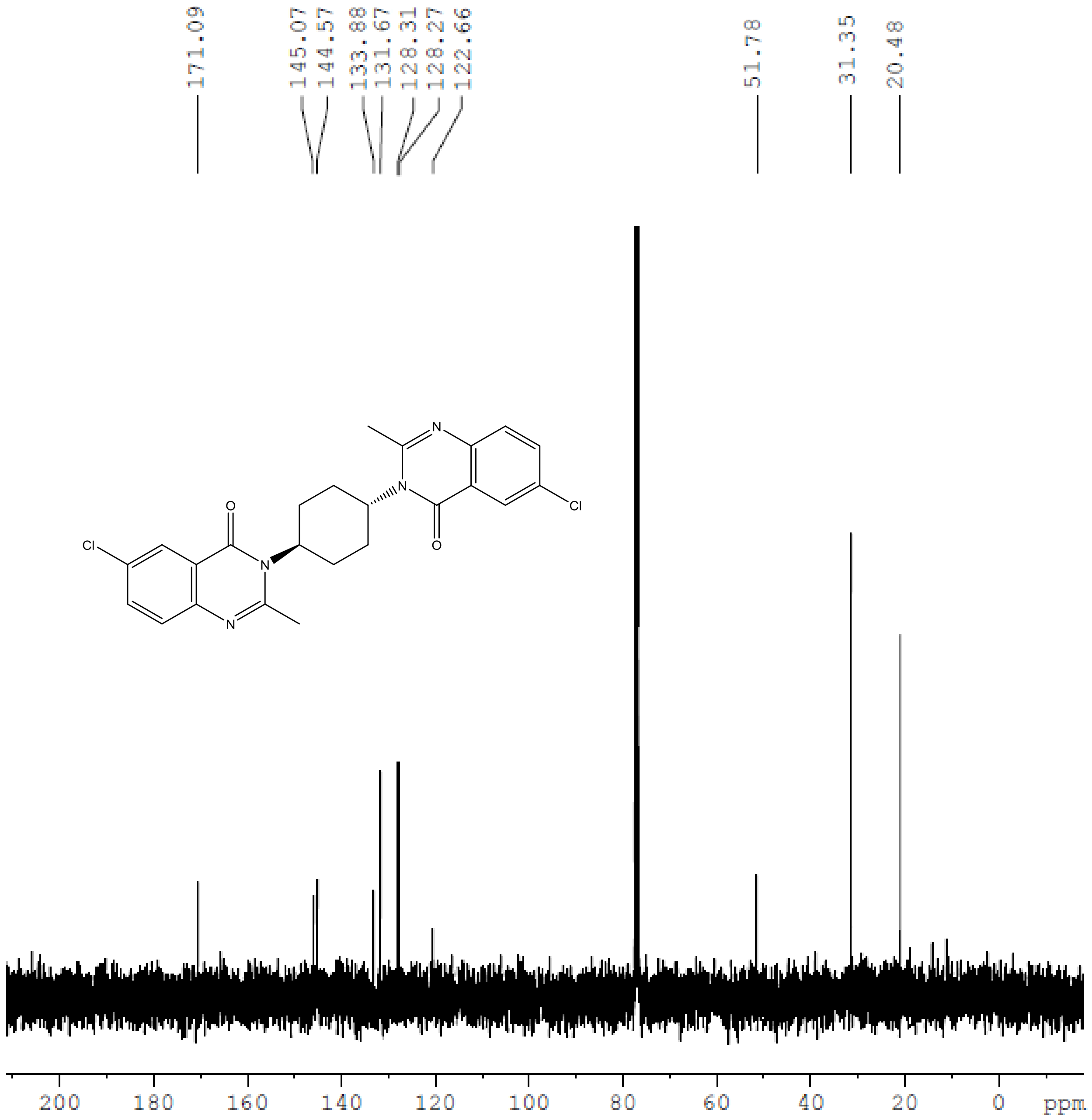
171.09
145.07
144.57
133.88
131.67
128.31
128.27
122.66
51.78
31.35
20.48
Cl
N
O
N
O
N
Cl
N
200
180
160
140
120
100
80
60
40
20
0
ppm

^{13}C NMR of **3c**

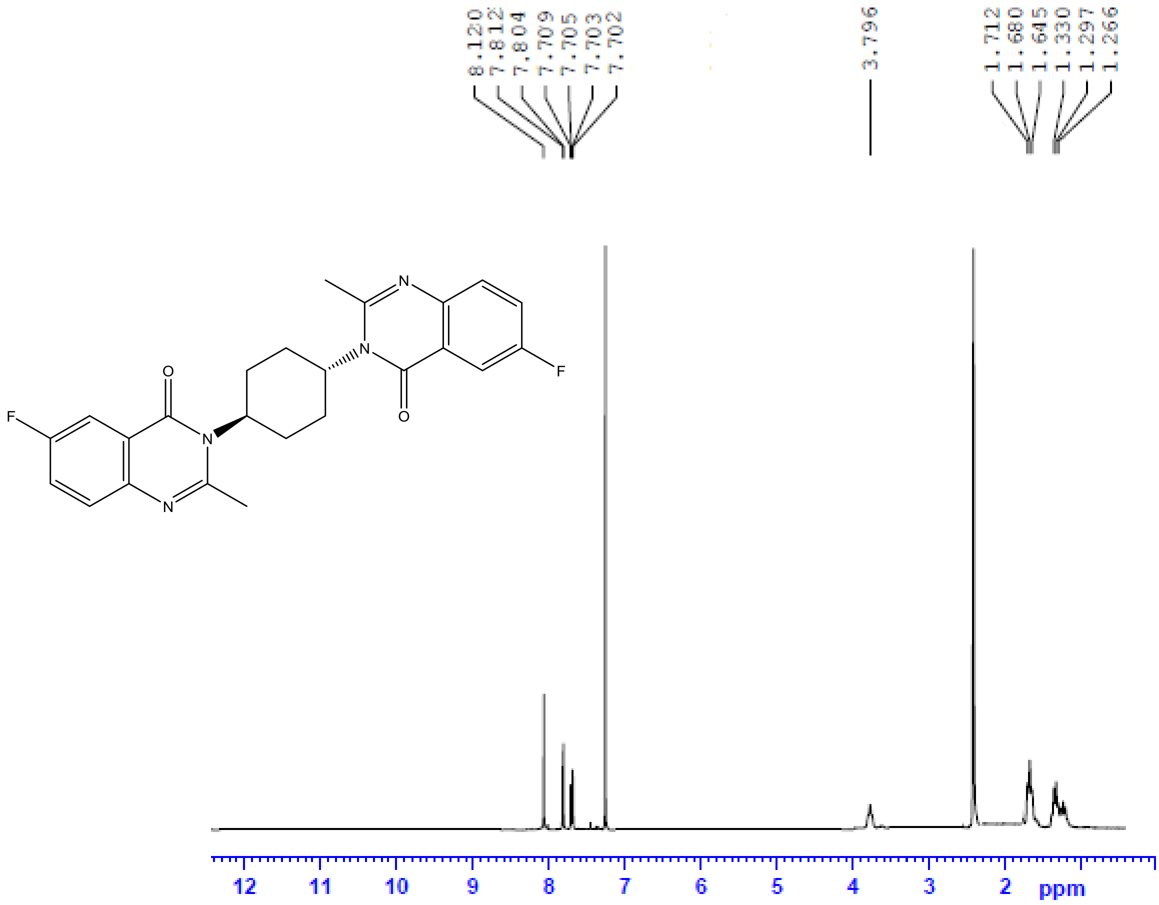
8.120
7.812
7.804
7.709
7.705
7.703
7.702
3.796
1.712
1.680
1.645
1.330
1.297
1.266
F
O
N
N
O
F
12
11
10
9
8
7
6
5
4
3
2
ppm

^{1}H NMR of **3d**

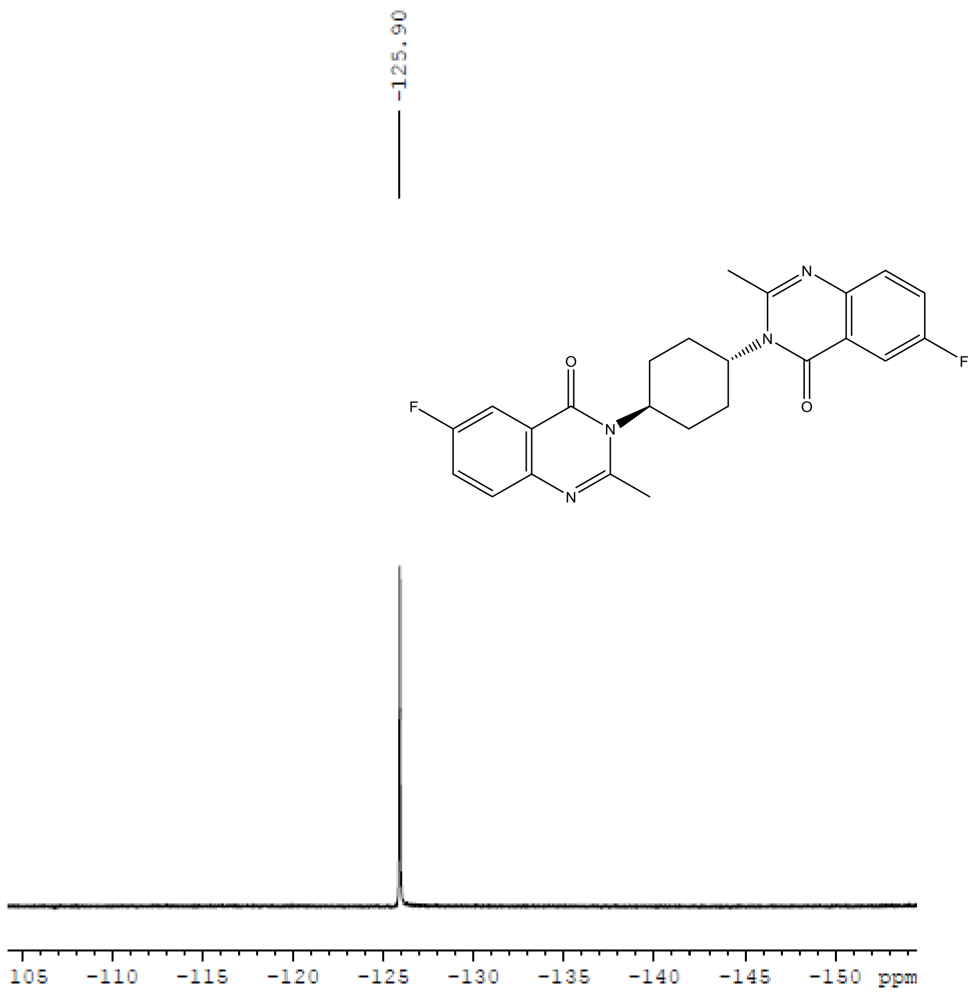

^{19}F NMR of **3d**

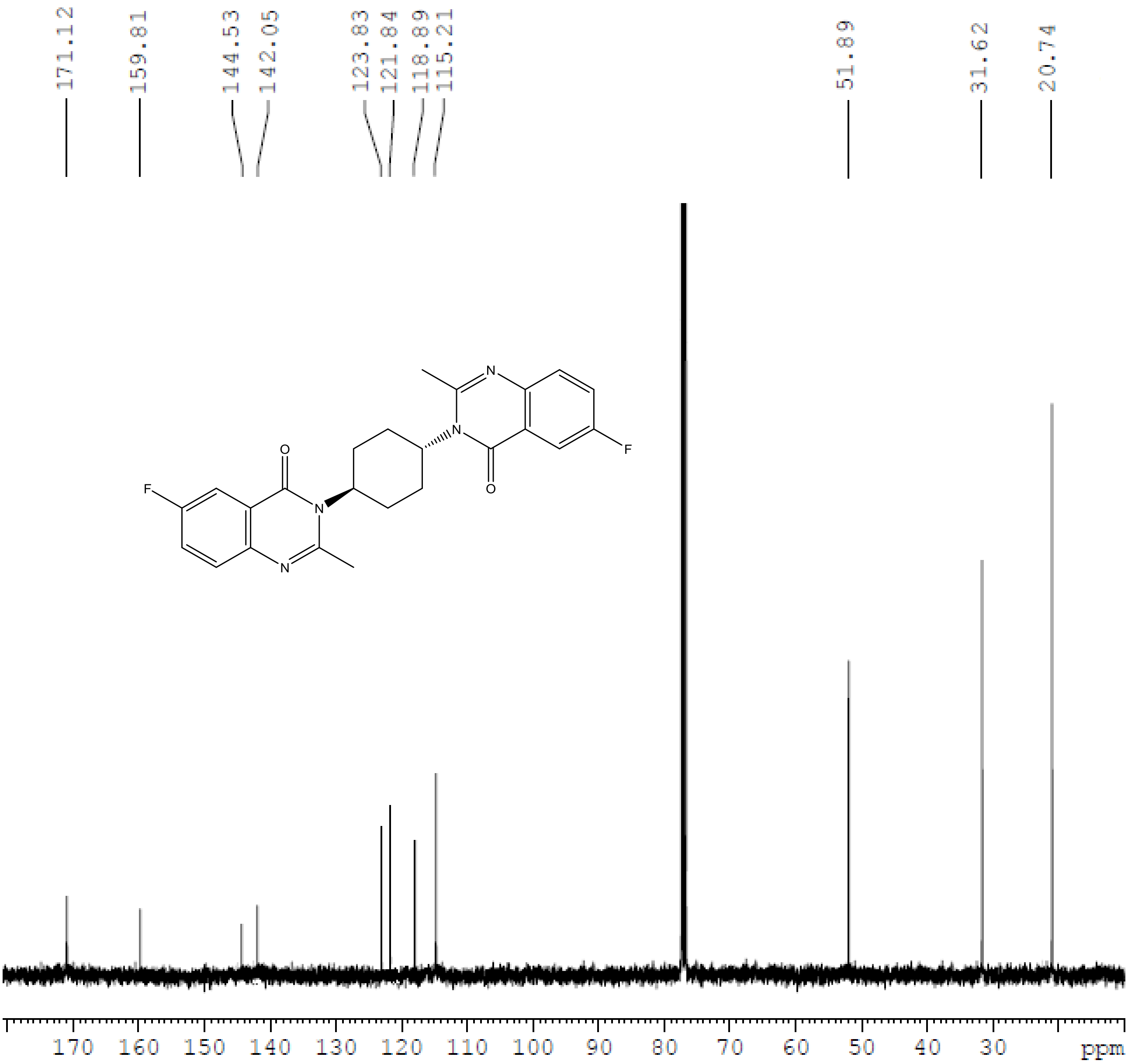
171.12
159.81
144.53
142.05
123.83
121.84
118.89
115.21
51.89
31.62
20.74
170
160
150
140
130
120
110
100
90
80
70
60
50
40
30
ppm
F
O
N
N
N
N
O
F

^{12}C NMR of **3d**

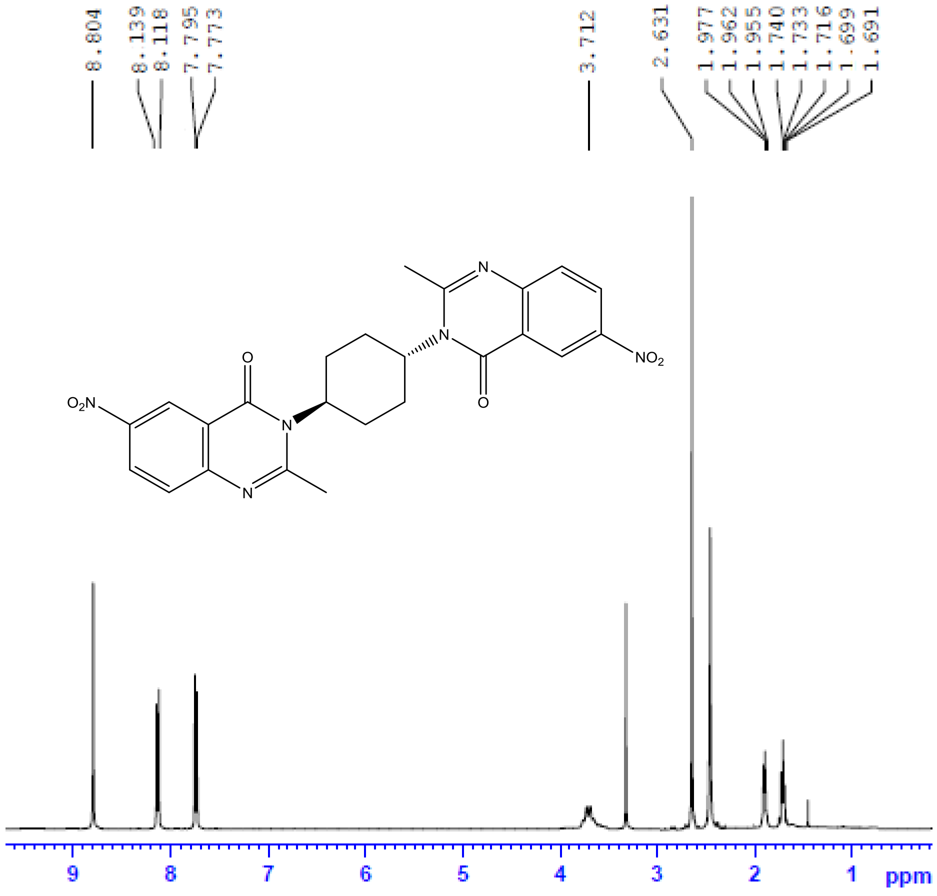
8.804
8.139
8.118
7.795
7.773
3.712
2.631
1.977
1.962
1.955
1.740
1.733
1.716
1.699
1.691
O₂N
N
O
N
NO₂
9
8
7
6
5
4
3
2
1
ppm

^{1}H NMR of **3e**

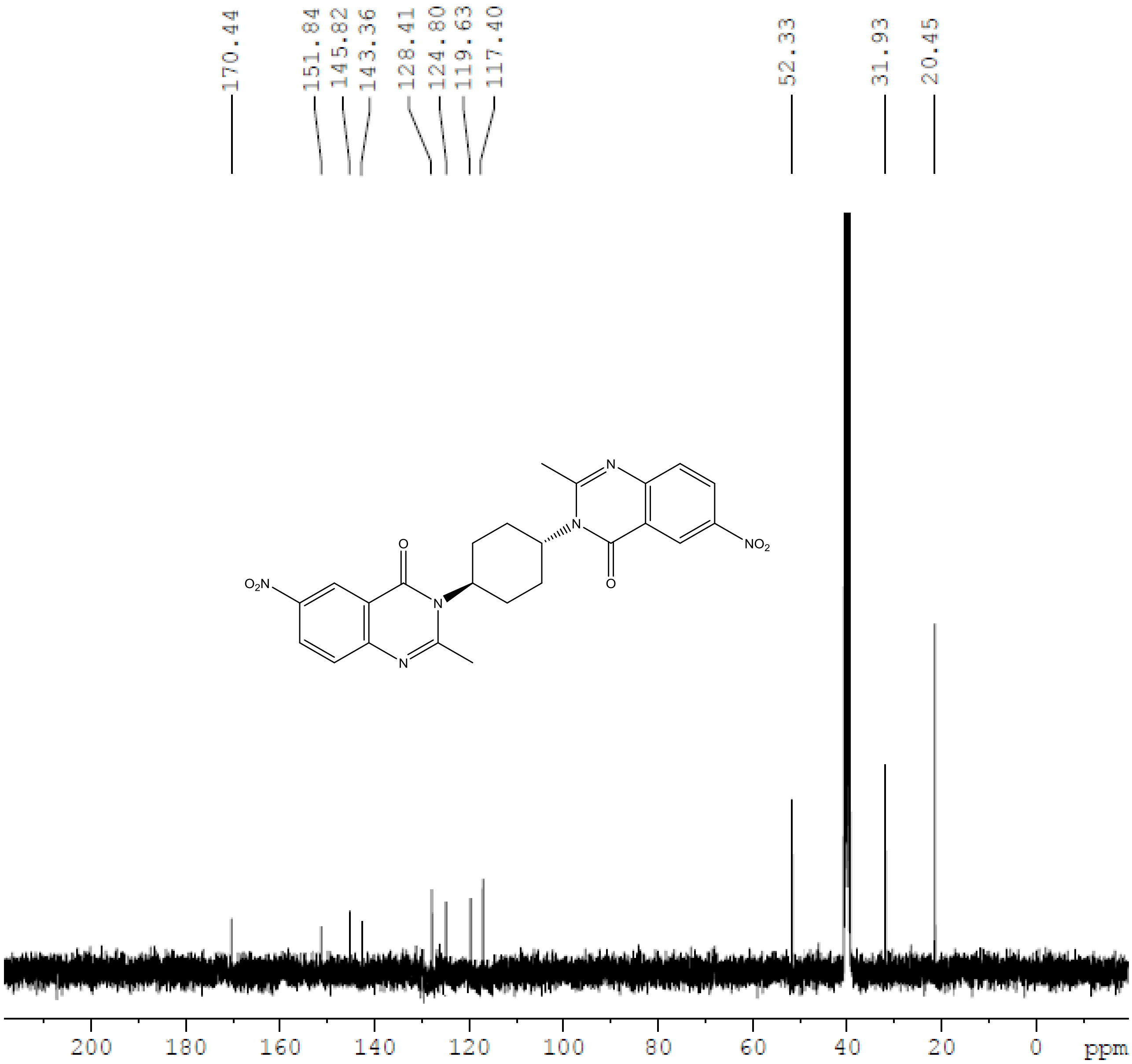
170.44
151.84
145.82
143.36
128.41
124.80
119.63
117.40
52.33
31.93
20.45
O
O₂N
N
N
N
N
NO₂
O
200
180
160
140
120
100
80
60
40
20
0
ppm

^{13}C NMR of **3e**

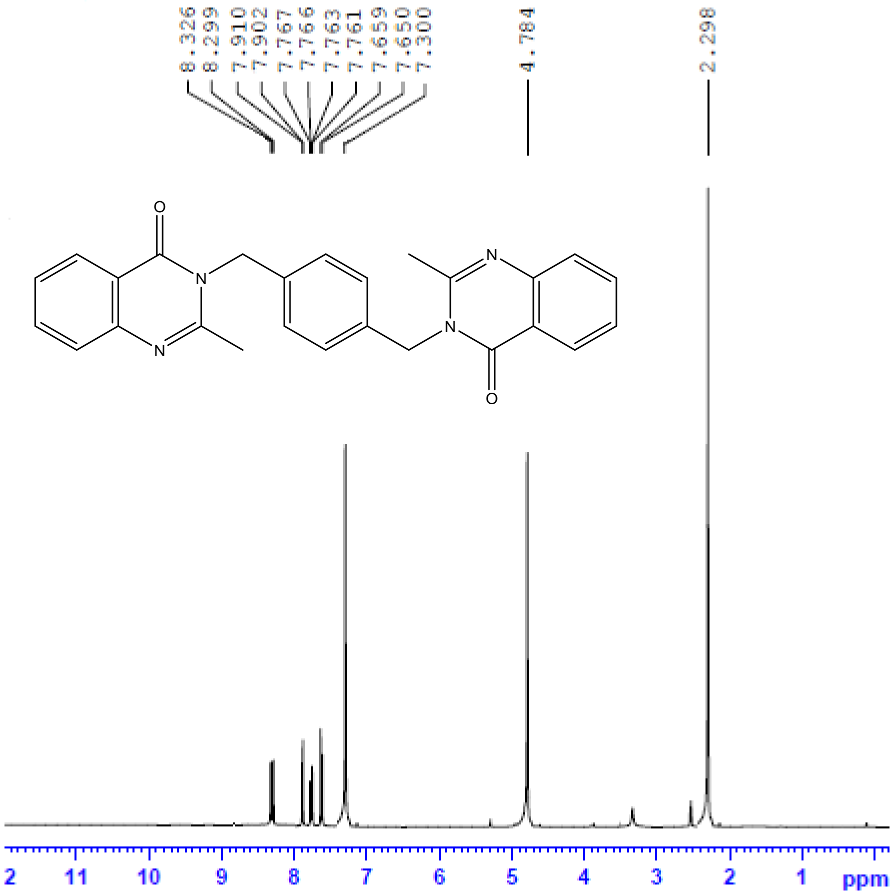

8.326
8.299
7.910
7.902
7.767
7.766
7.763
7.761
7.659
7.650
7.300
4.784
2.298
O
N
N
N
N
O
2
11
10
9
8
7
6
5
4
3
2
1
ppm

^{1}H NMR of **4a**

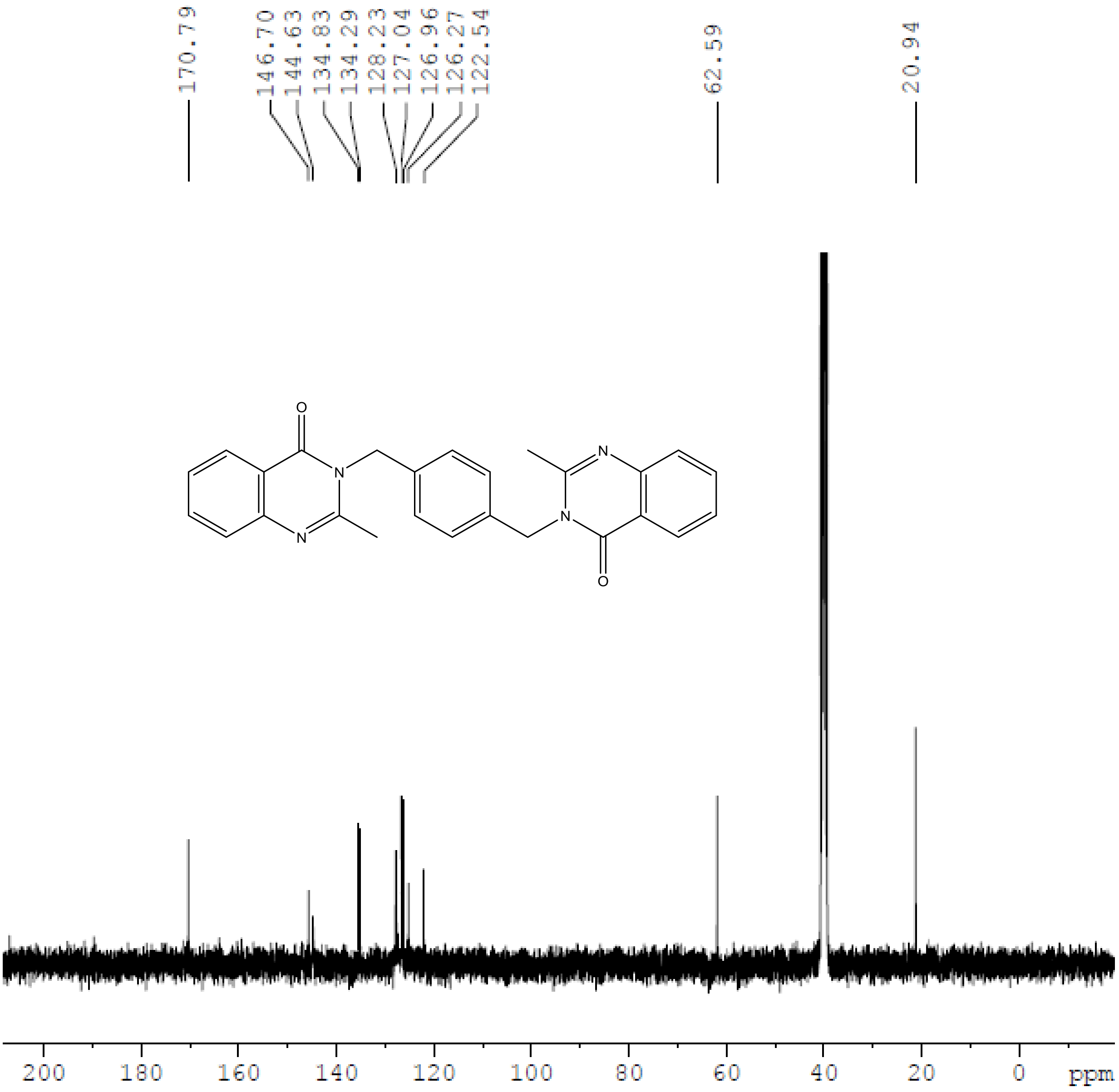

170.79
146.70
144.63
134.83
134.29
128.23
127.04
126.96
126.27
122.54
62.59
20.94
O
N
N
N
N
O
200
180
160
140
120
100
80
60
40
20
0
ppm

^{13}C NMR of **4a**

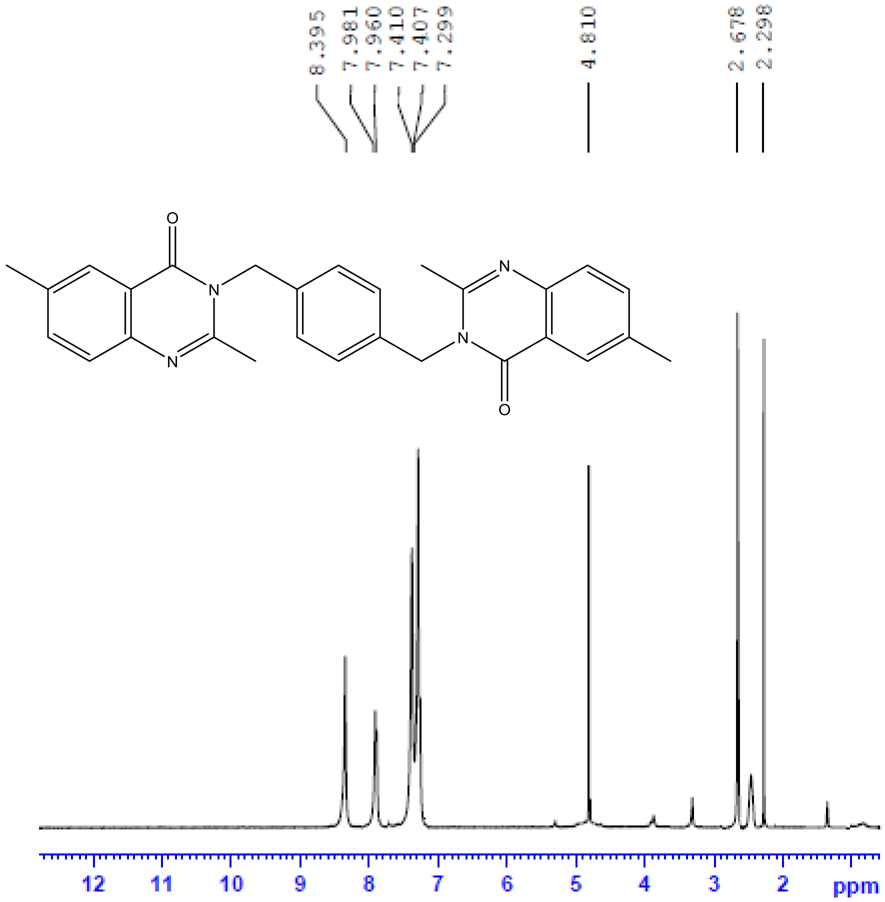
8.395
7.981
7.960
7.410
7.407
7.299
4.810
2.678
2.298
12
11
10
9
8
7
6
5
4
3
2
ppm

^{1}H NMR of **4b**

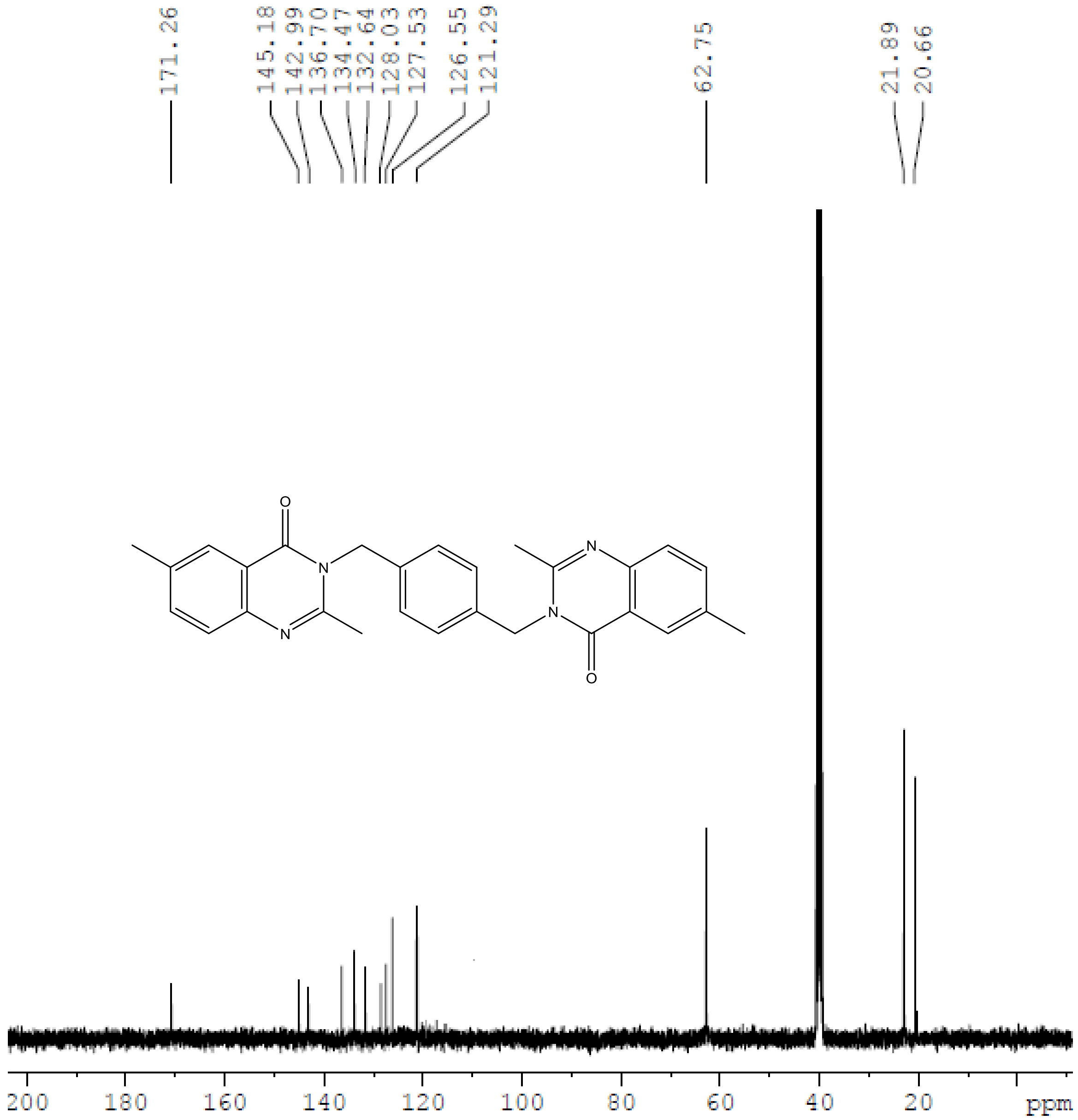
171.26
145.18
142.99
136.70
134.47
132.64
128.03
127.53
126.55
121.29
62.75
21.89
20.66
O
N
N
O
200 180 160 140 120 100 80 60 40 20 ppm

^{13}C NMR of **4b**

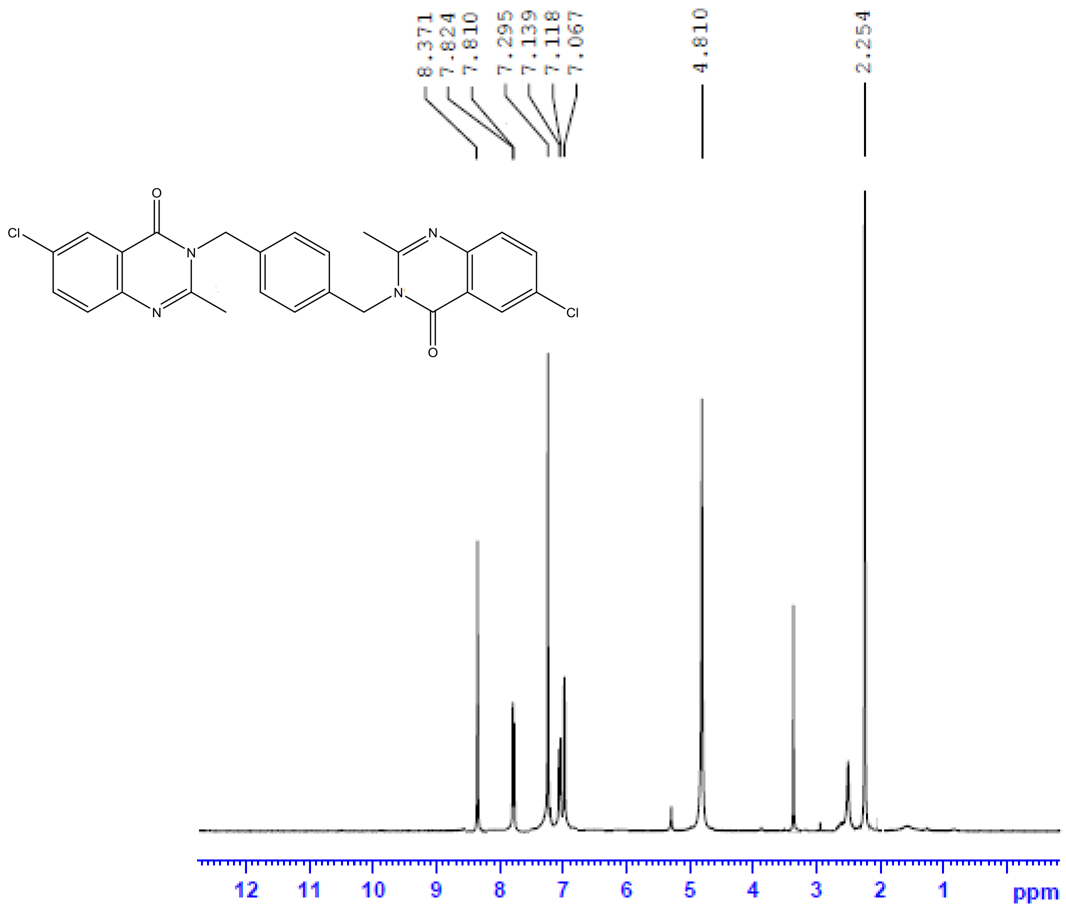
8.371
7.824
7.810
7.295
7.139
7.118
7.067
4.810
2.254
O
Cl
N
N
N
N
Cl
O
12
11
10
9
8
7
6
5
4
3
2
1
ppm

^{1}H NMR of **4c**

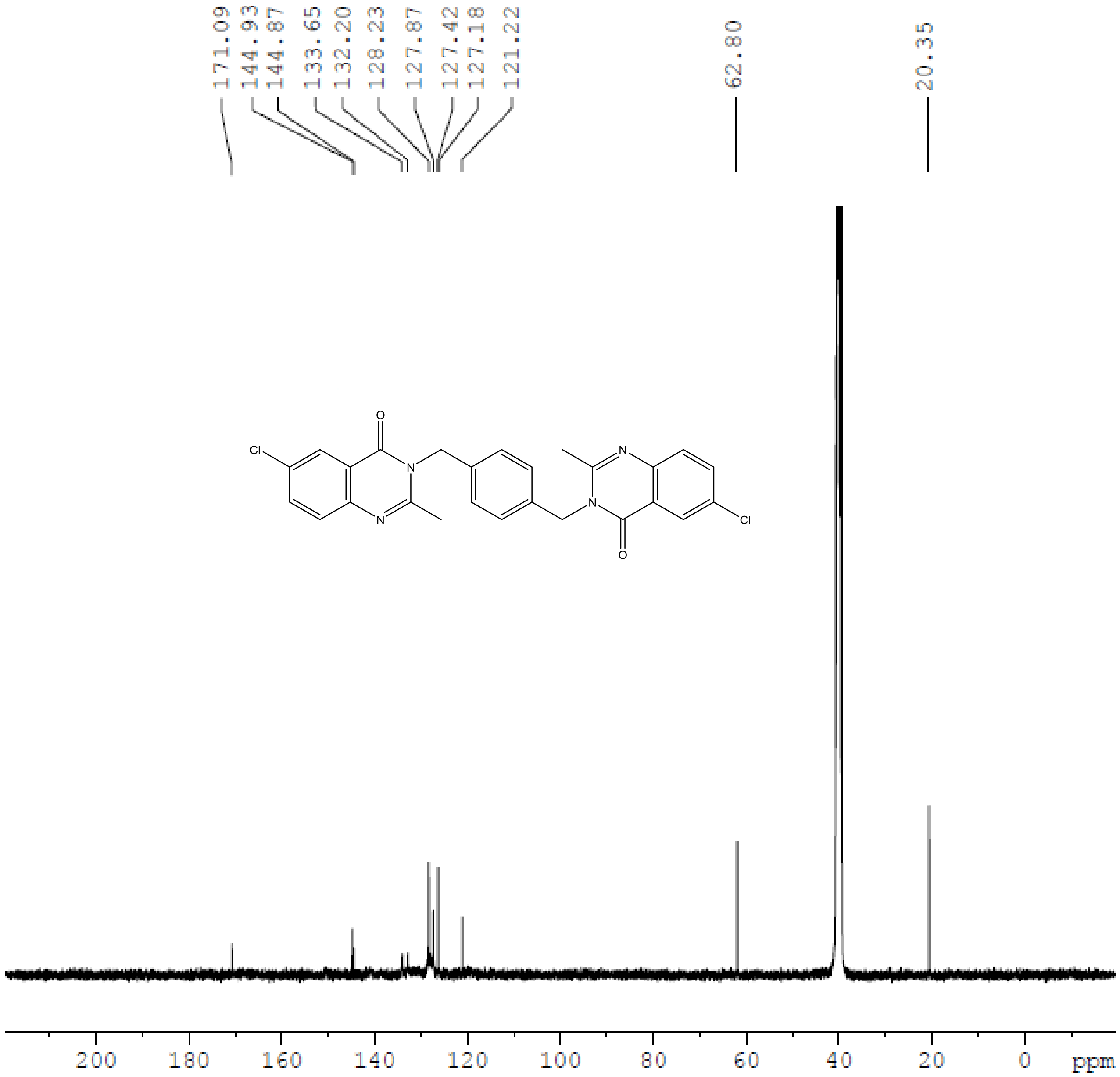
171.09
144.93
144.87
133.65
132.20
128.23
127.87
127.42
127.18
121.22
62.80
20.35
O
Cl
N
N
N
N
Cl
O
200
180
160
140
120
100
80
60
40
20
0
ppm

^{13}C NMR of **4c**

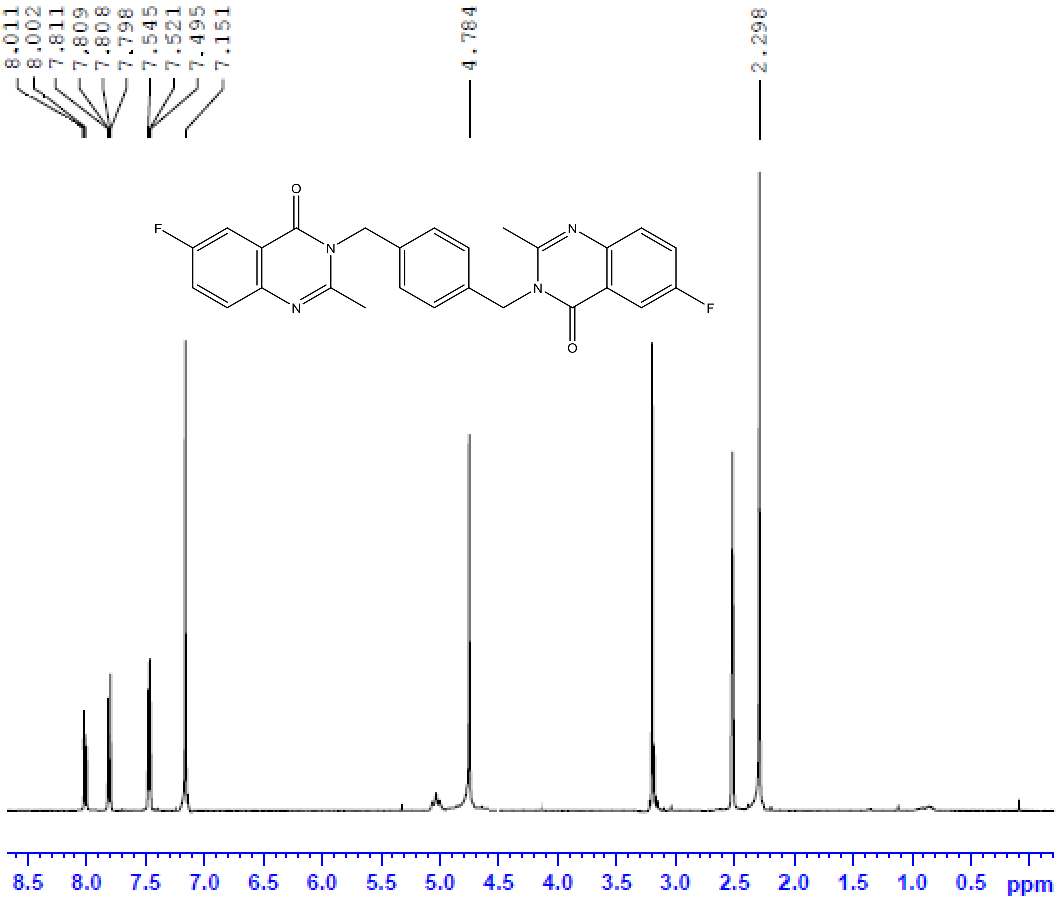
8.011
8.002
7.811
7.809
7.808
7.798
7.545
7.521
7.495
7.151
4.784
2.298
O
F
N
N
N
N
F
O
8.5
8.0
7.5
7.0
6.5
6.0
5.5
5.0
4.5
4.0
3.5
3.0
2.5
2.0
1.5
1.0
0.5
ppm

^{1}H NMR of **4d**

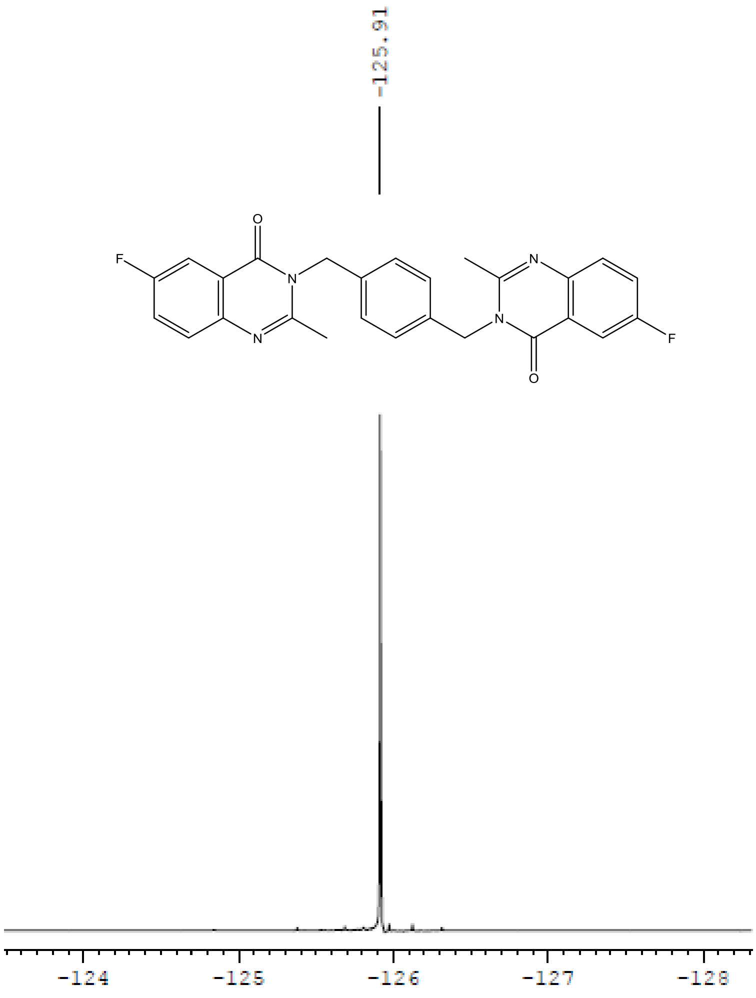

^{19}F NMR of **4d**

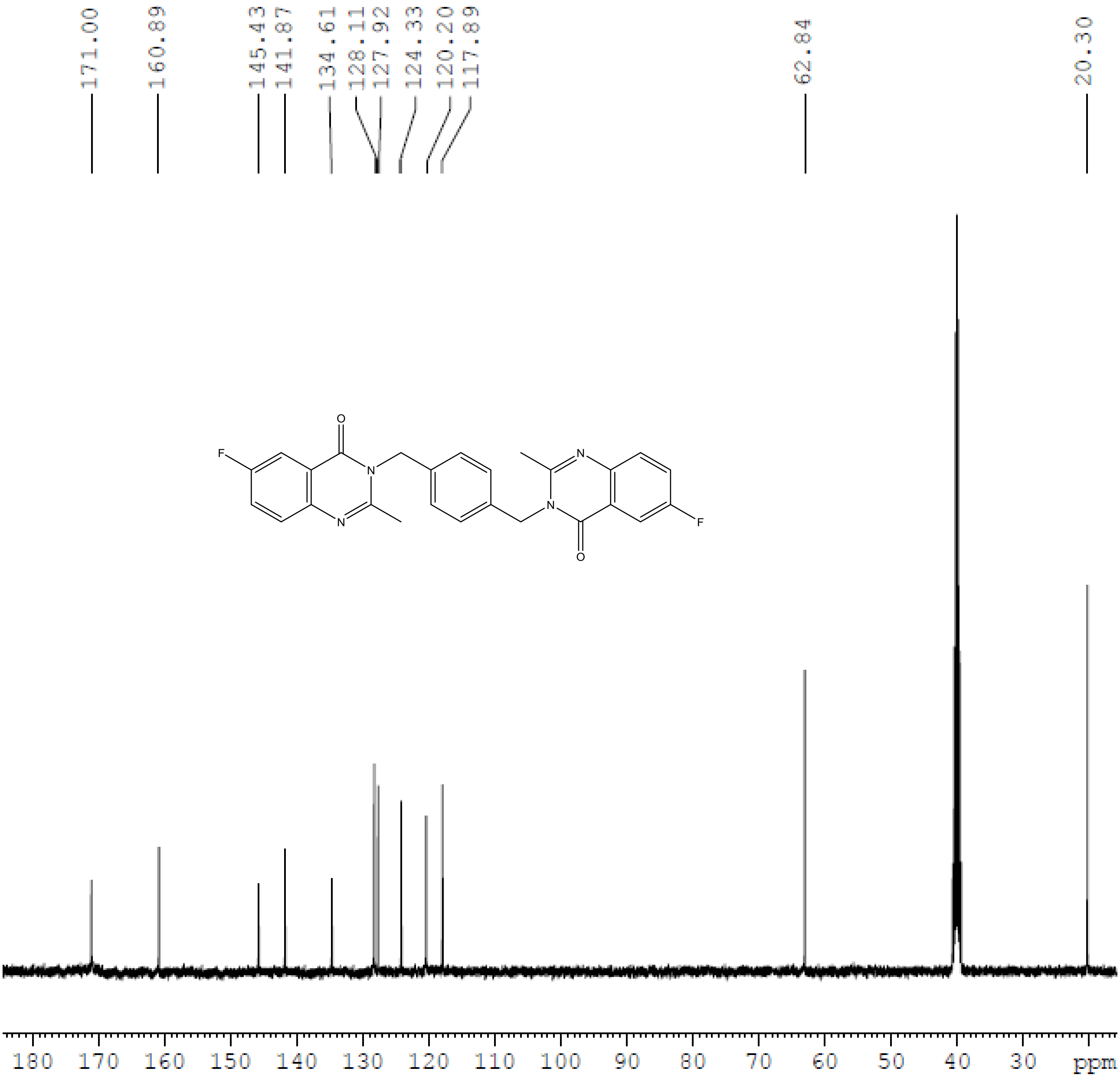
171.00
160.89
145.43
141.87
134.61
128.11
127.92
124.33
120.20
117.89
62.84
20.30
O
F
N
N
N
N
O
F
180
170
160
150
140
130
120
110
100
90
80
70
60
50
40
30
ppm

^{13}C NMR of **4d**

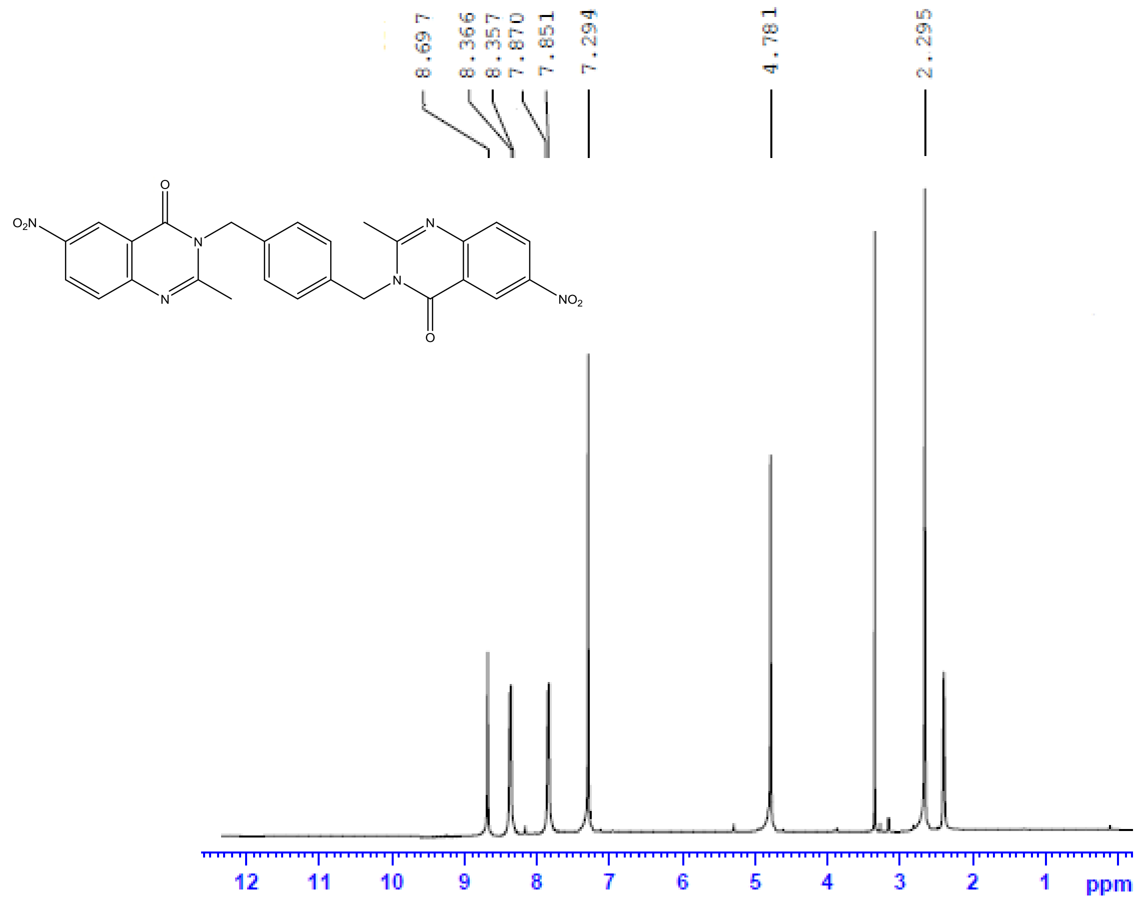

8.697
8.366
8.357
7.870
7.851
7.294
4.781
2.295
O
O_2N
N
N
N
N
NO_2
O
12
11
10
9
8
7
6
5
4
3
2
1
ppm

^{1}H NMR of **4e**

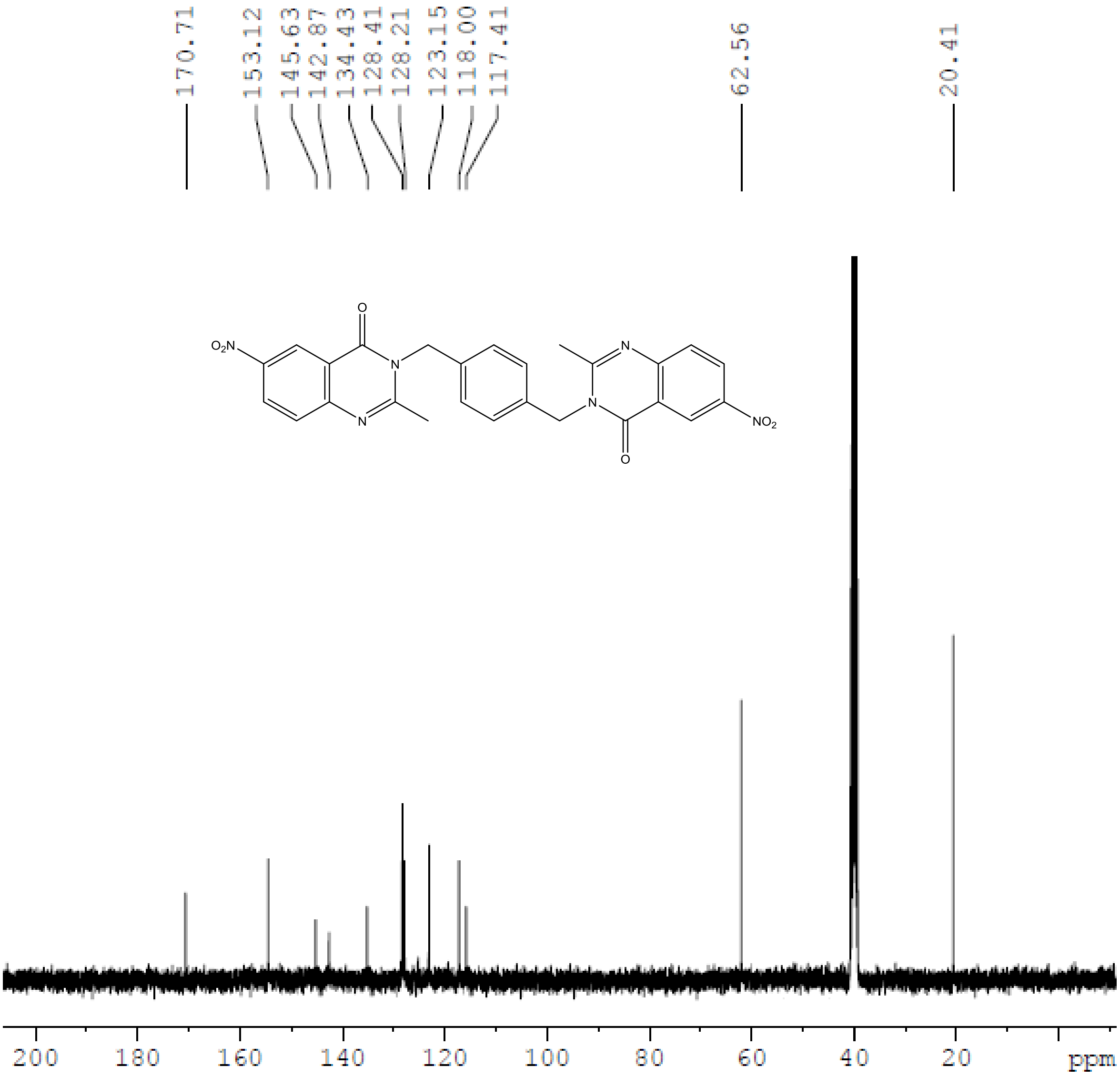
170.71
153.12
145.63
142.87
134.43
128.41
128.21
123.15
118.00
117.41
62.56
20.41
O
O_2N
N
N
N
N
O
NO_2
200
180
160
140
120
100
80
60
40
20
ppm

^{13}C NMR of **4e**

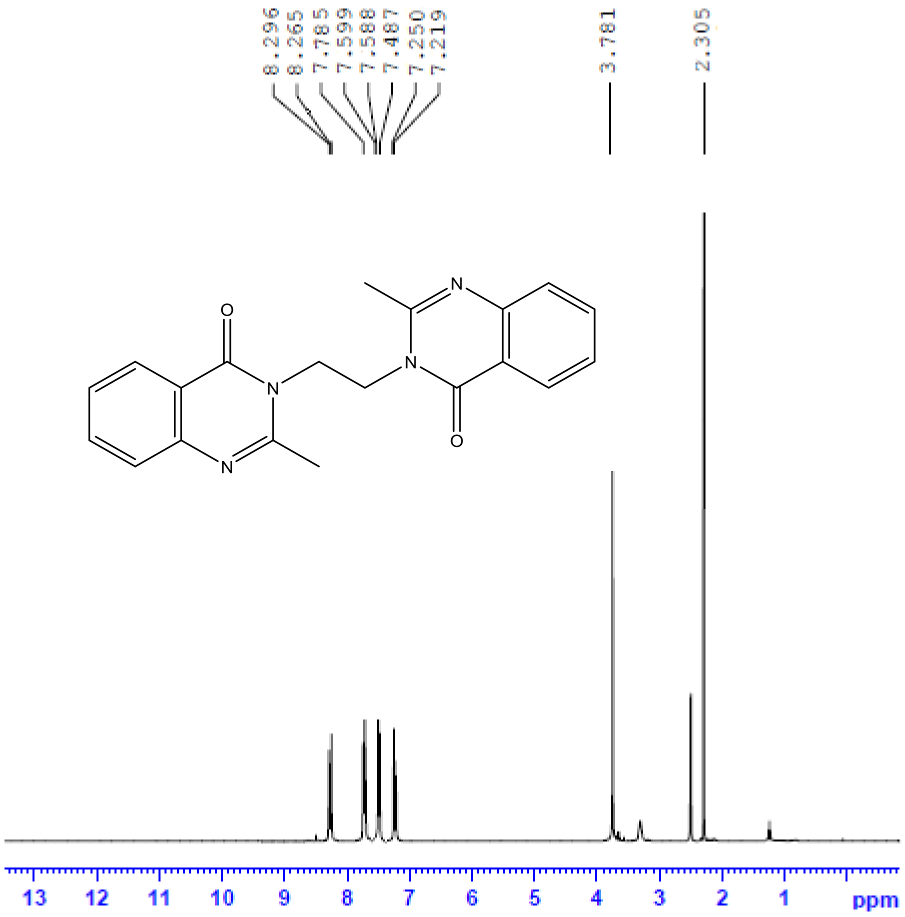
8.296
8.265
7.785
7.599
7.588
7.487
7.250
7.219
3.781
2.305
O
N
N
N
N
O
13
12
11
10
9
8
7
6
5
4
3
2
1
ppm

^{1}H NMR of **5a**

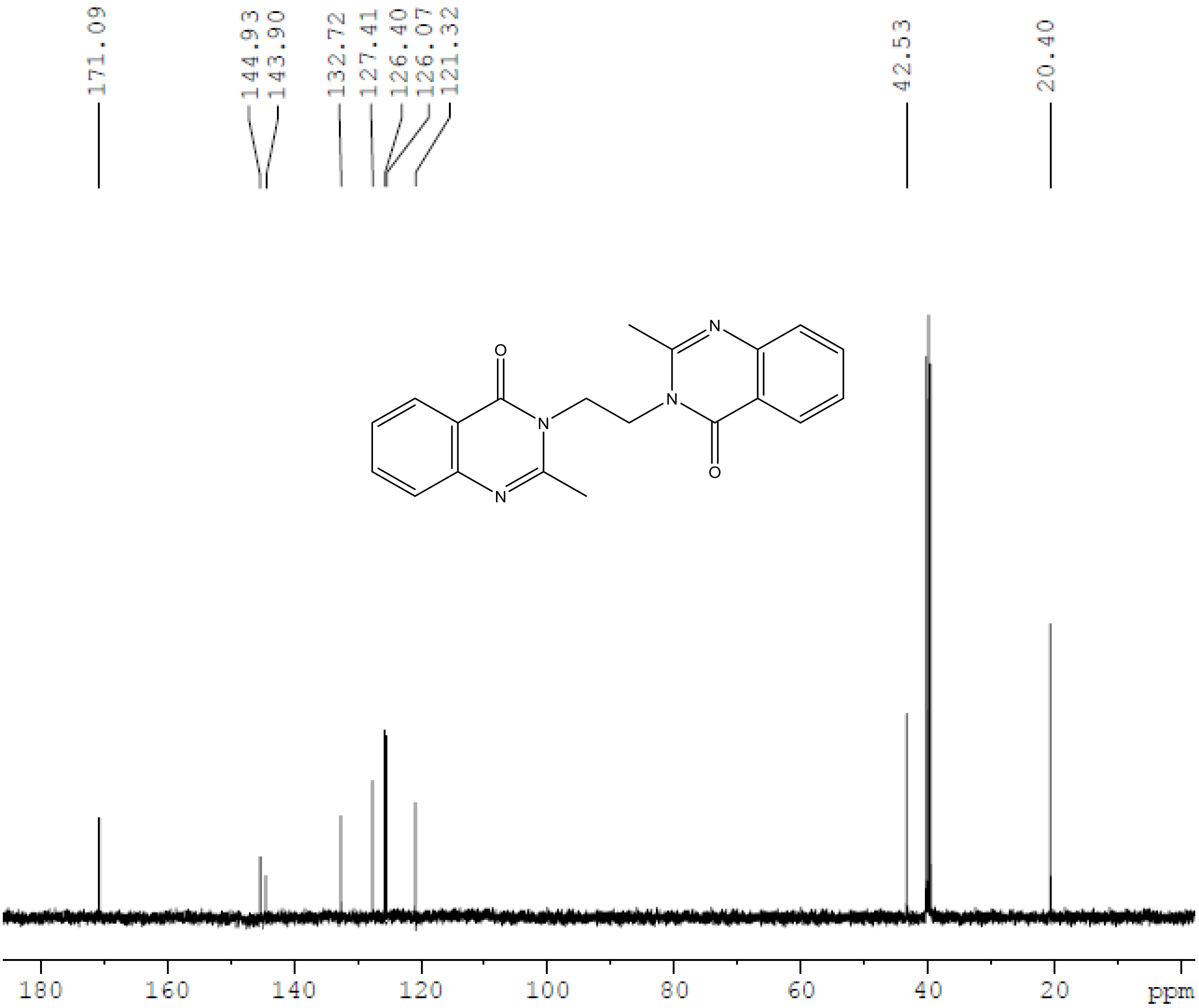
171.09
144.93
143.90
132.72
127.41
126.40
126.07
121.32
42.53
20.40
O
N
N
N
N
O
180
160
140
120
100
80
60
40
20
ppm

^{13}C NMR of **5a**

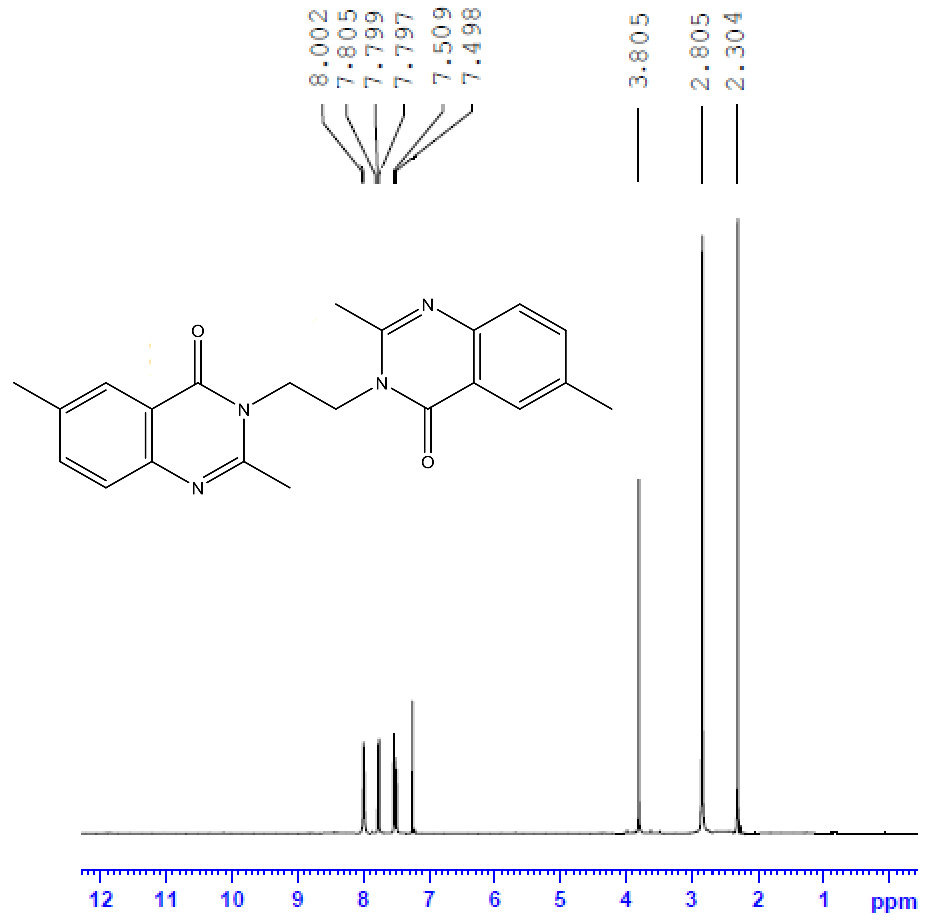

8.002
7.805
7.799
7.797
7.509
7.498
3.805
2.805
2.304
12
11
10
9
8
7
6
5
4
3
2
1
ppm

^{1}H NMR of **5b**

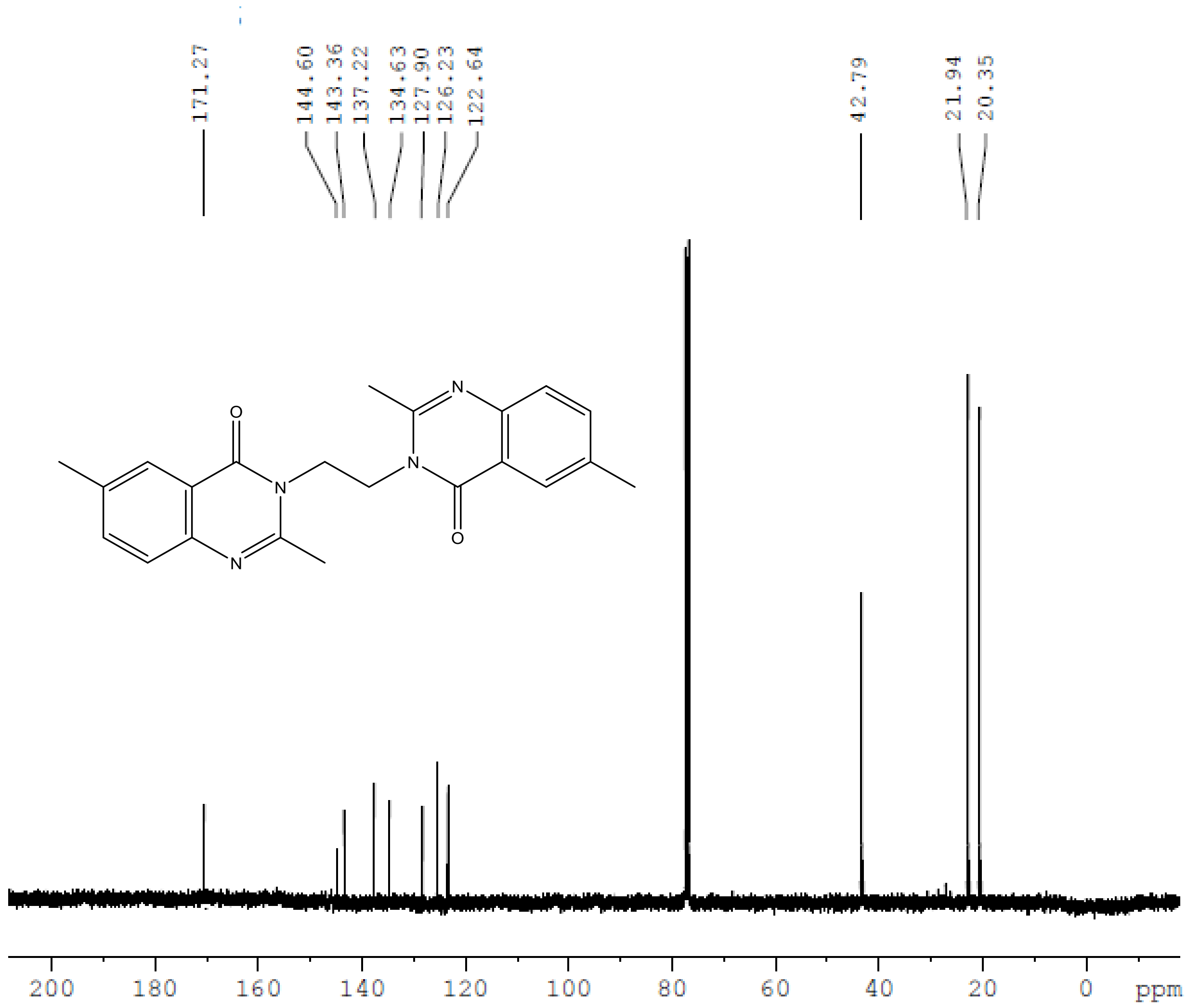
171.27
144.60
143.36
137.22
134.63
127.90
126.23
122.64
42.79
21.94
20.35
O
N
N
N
N
O
200
180
160
140
120
100
80
60
40
20
0
ppm

^{13}C NMR of **5b**

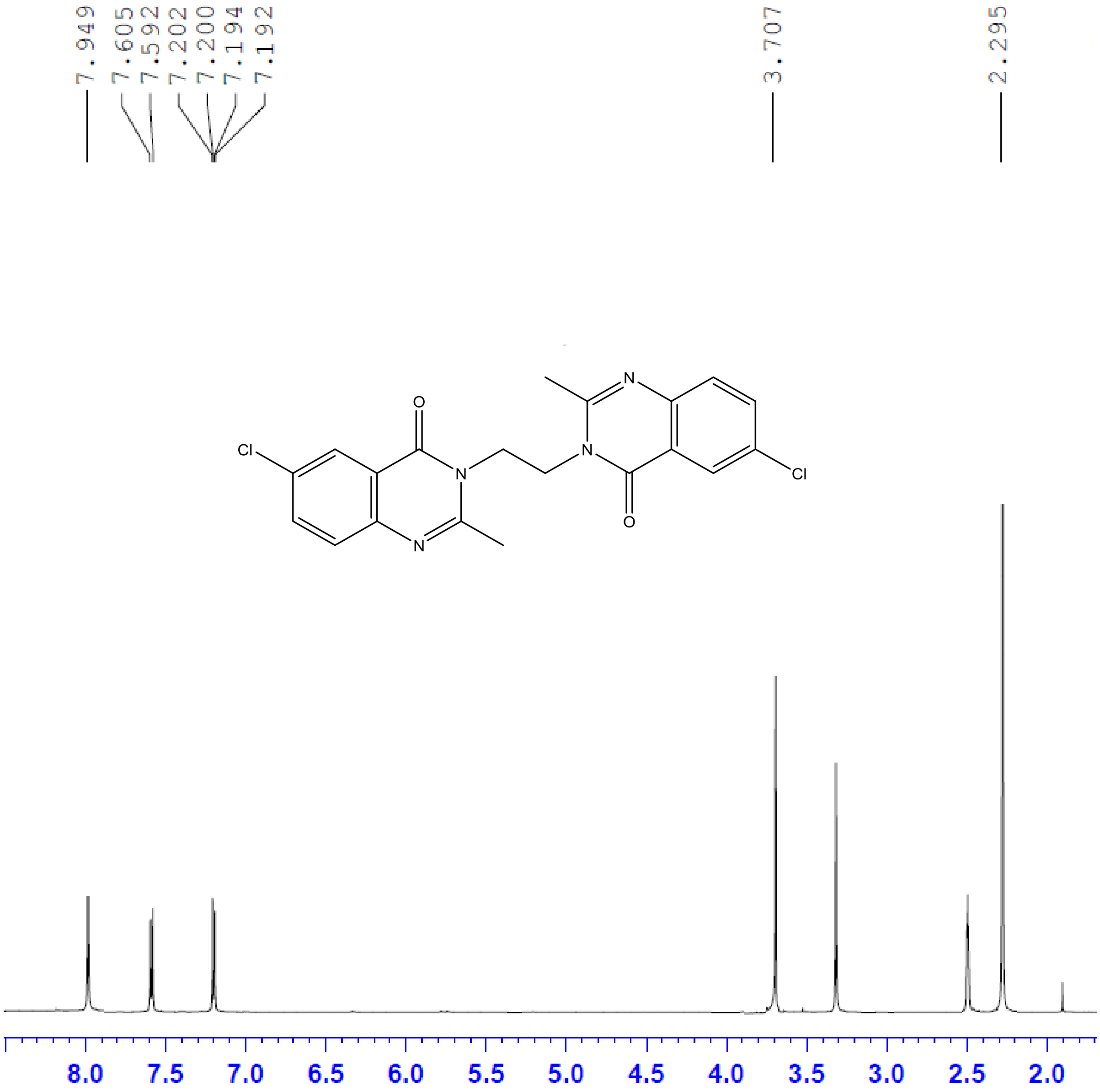
7.949
7.605
7.592
7.202
7.200
7.194
7.192
3.707
2.295
Cl
O
N
N
N
N
O
Cl
8.0
7.5
7.0
6.5
6.0
5.5
5.0
4.5
4.0
3.5
3.0
2.5
2.0

^{1}H NMR of **5c**

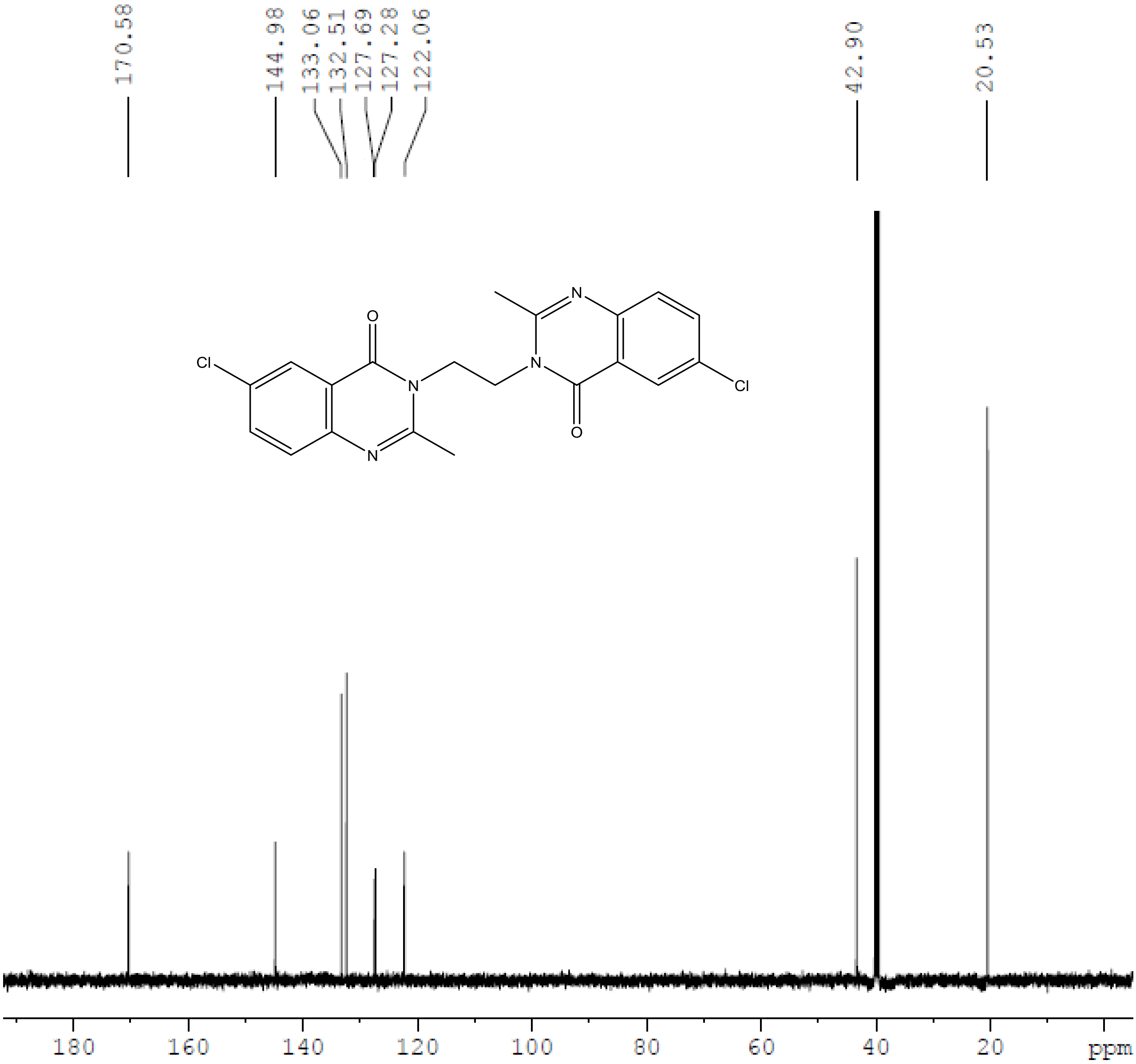
170.58
144.98
133.06
132.51
127.69
127.28
122.06
42.90
20.53
O
N
Cl
N
N
N
Cl
O
180
160
140
120
100
80
60
40
20
ppm

^{13}C NMR of **5c**

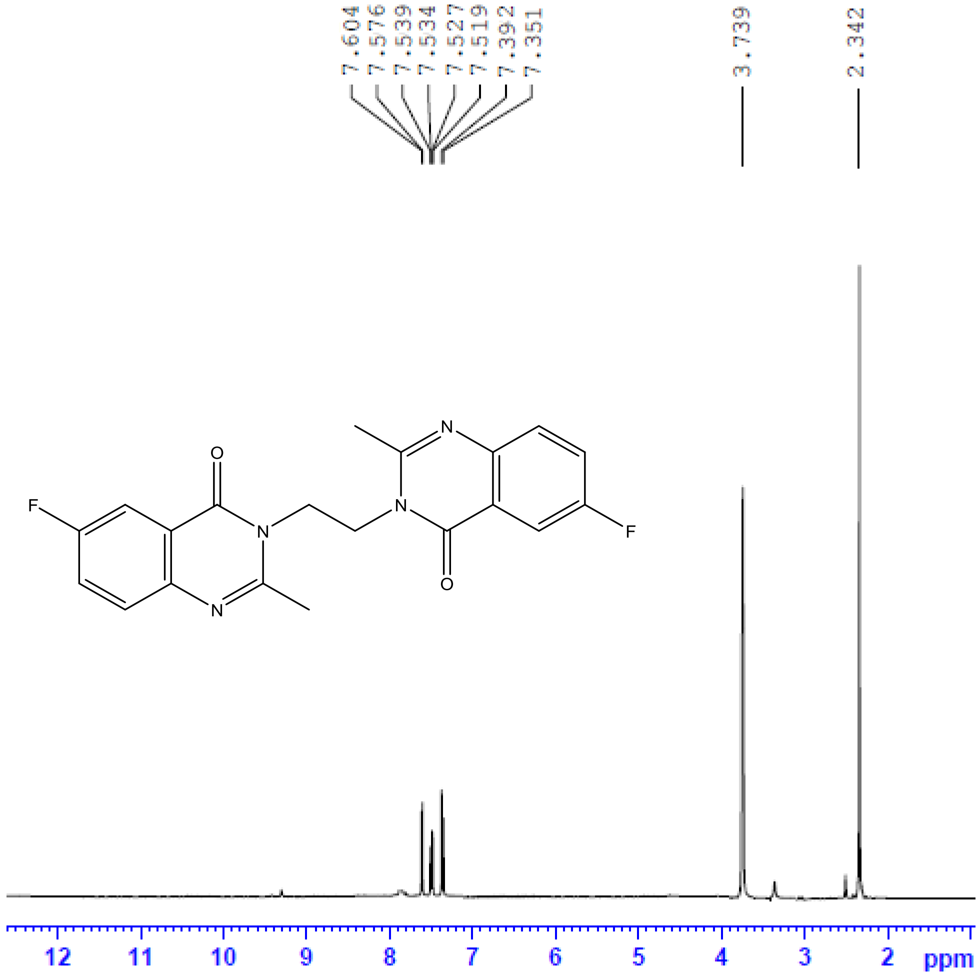
7.604
7.576
7.539
7.534
7.527
7.519
7.392
7.351
3.739
2.342
F
O
N
N
N
N
O
F
12
11
10
9
8
7
6
5
4
3
2
ppm

^{1}H NMR of **5d**

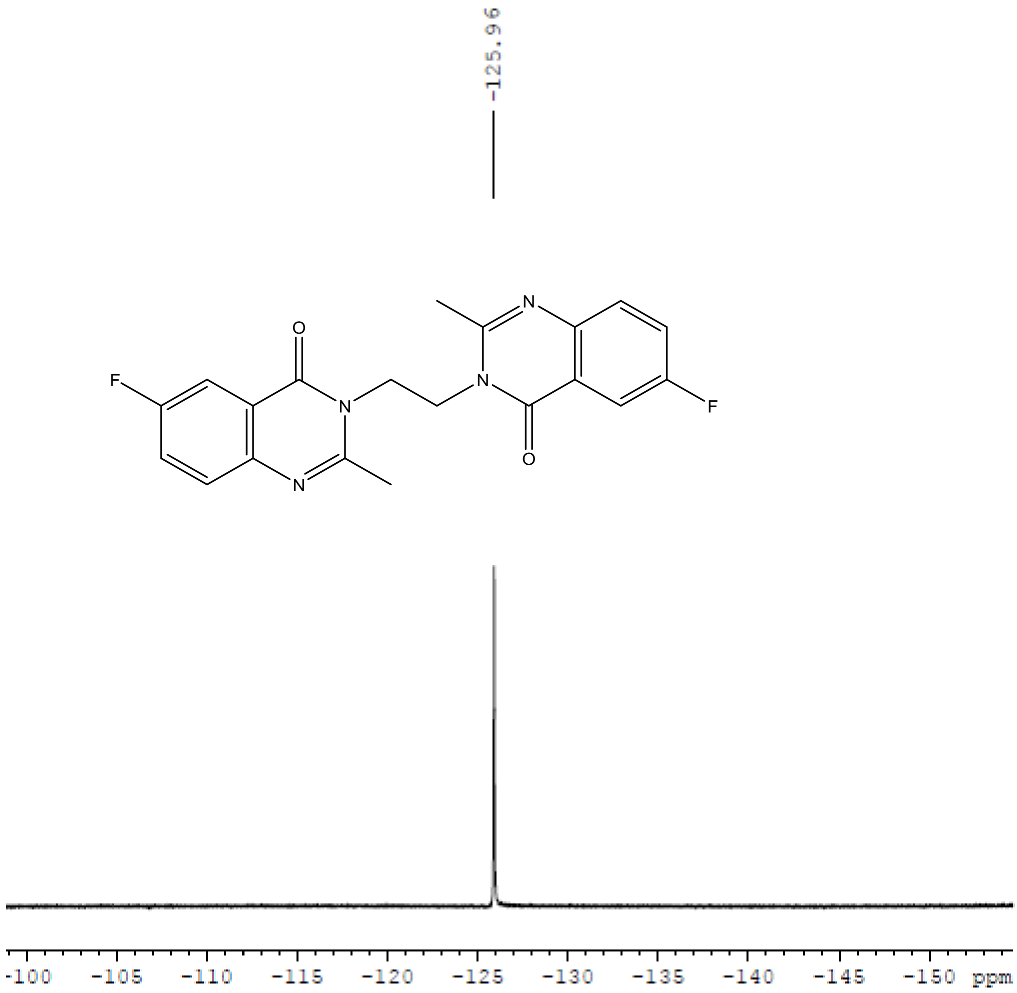

^{19}F NMR of **5d**

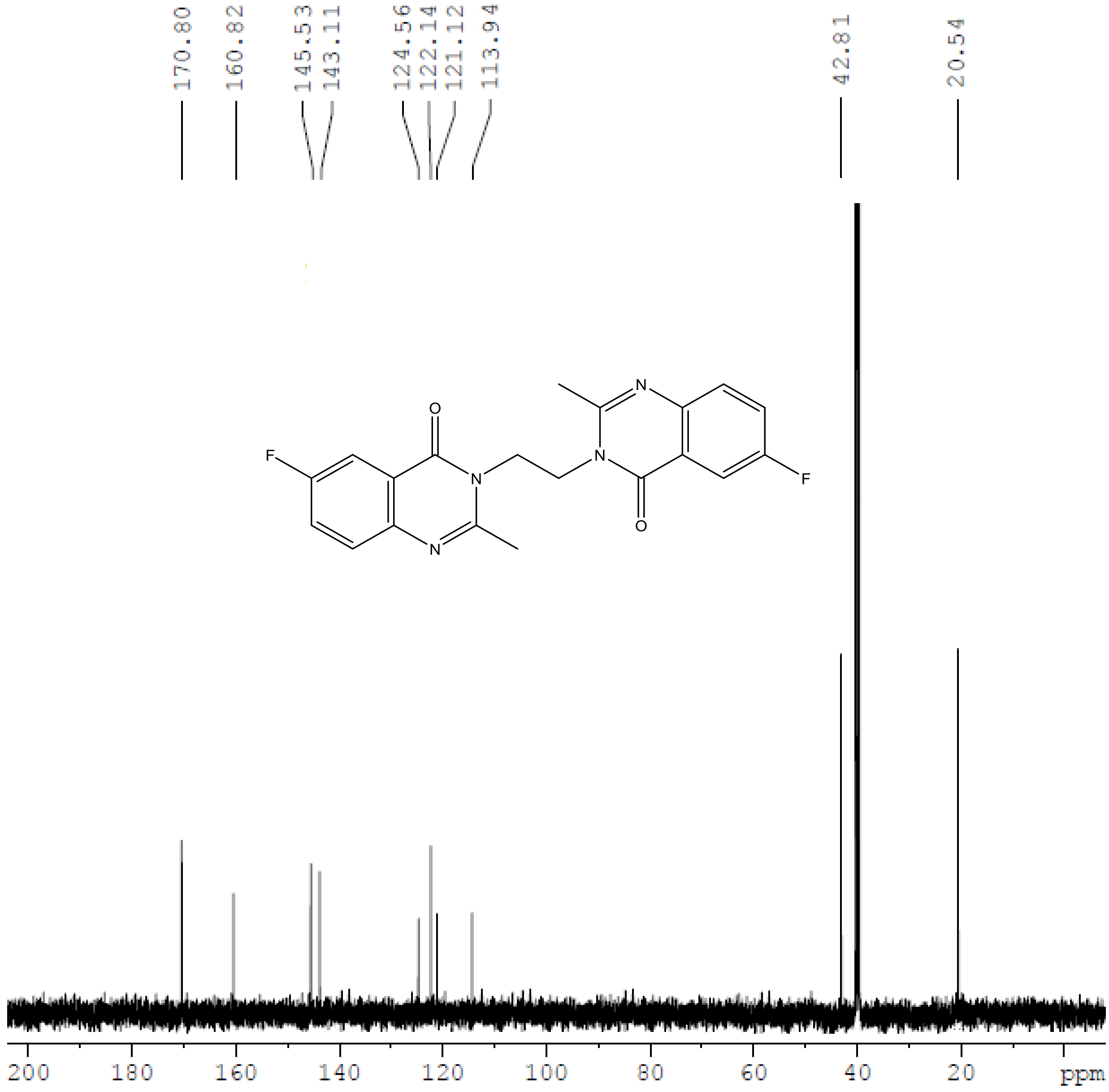
170.80
160.82
145.53
143.11
124.56
122.14
121.12
113.94
42.81
20.54
F
O
N
N
N
N
O
F
200
180
160
140
120
100
80
60
40
20
ppm

^{13}C NMR of **5d**

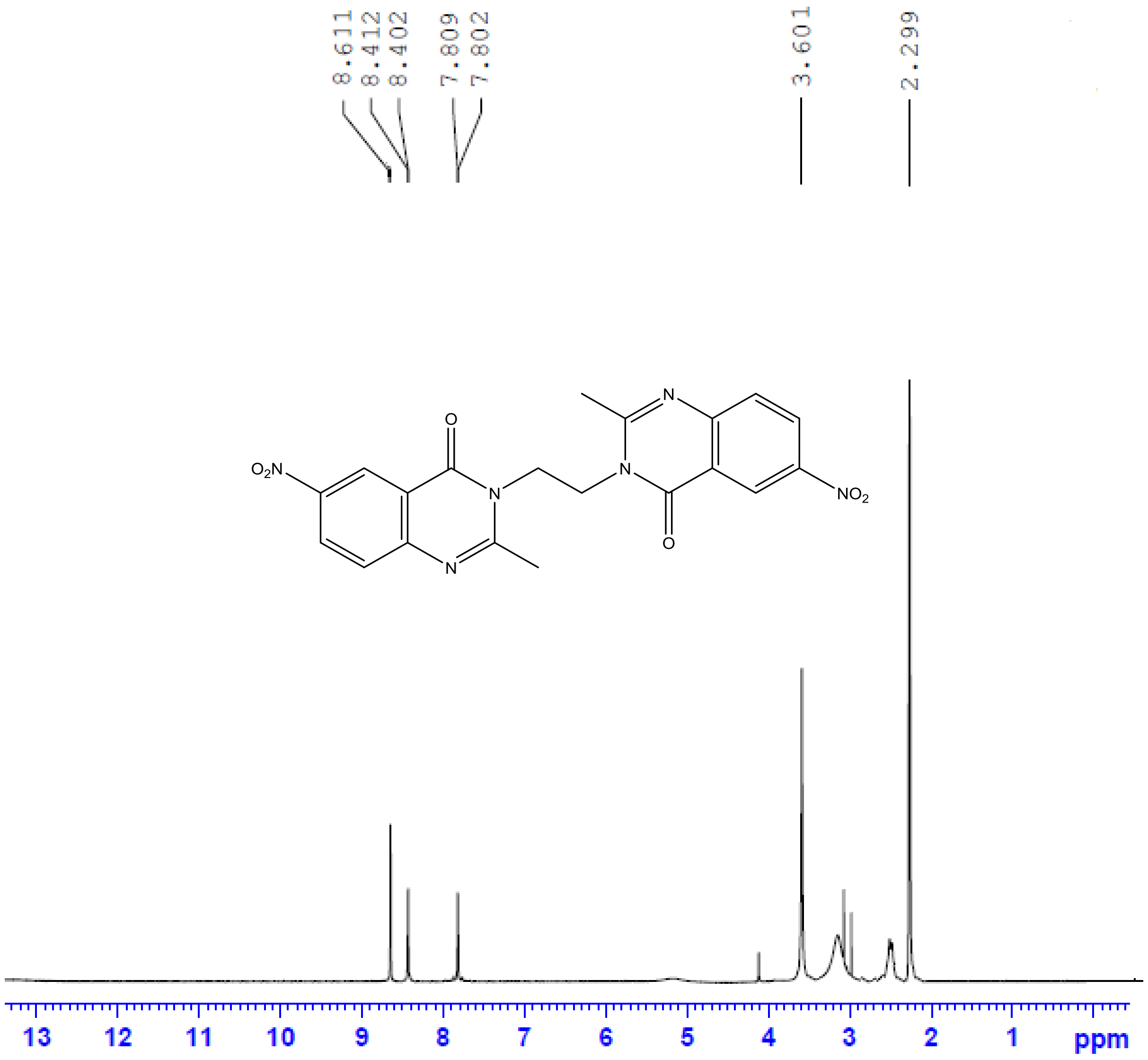
8.611
8.412
8.402
7.809
7.802
3.601
2.299
O
O2N
N
N
N
N
NO2
O
13
12
11
10
9
8
7
6
5
4
3
2
1
ppm

^{1}H NMR of **5e**

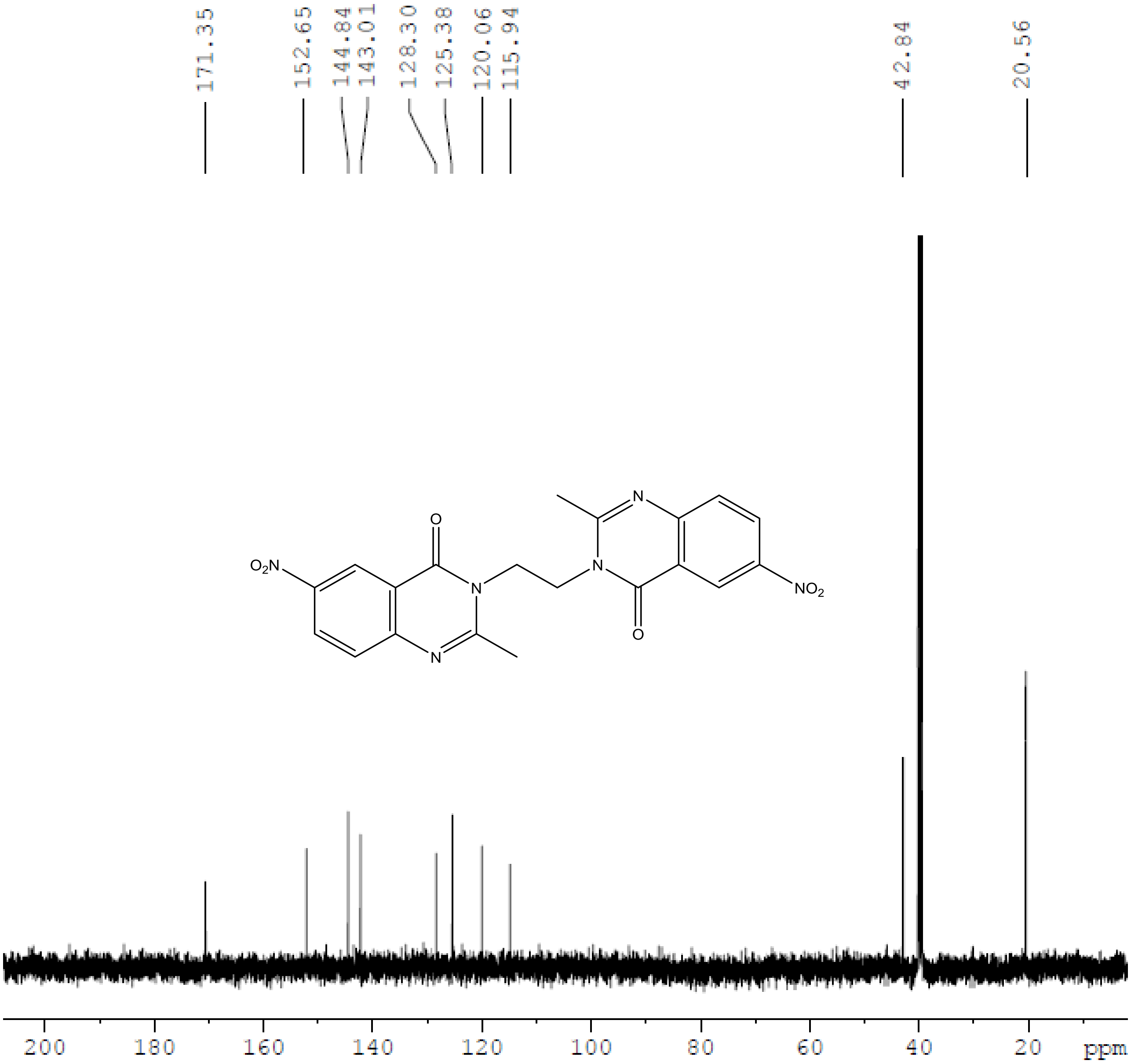
171.35
152.65
144.84
143.01
128.30
125.38
120.06
115.94
42.84
20.56
O
O2N
N
N
N
N
NO2
O
200
180
160
140
120
100
80
60
40
20
ppm

^{13}C NMR of **5e**

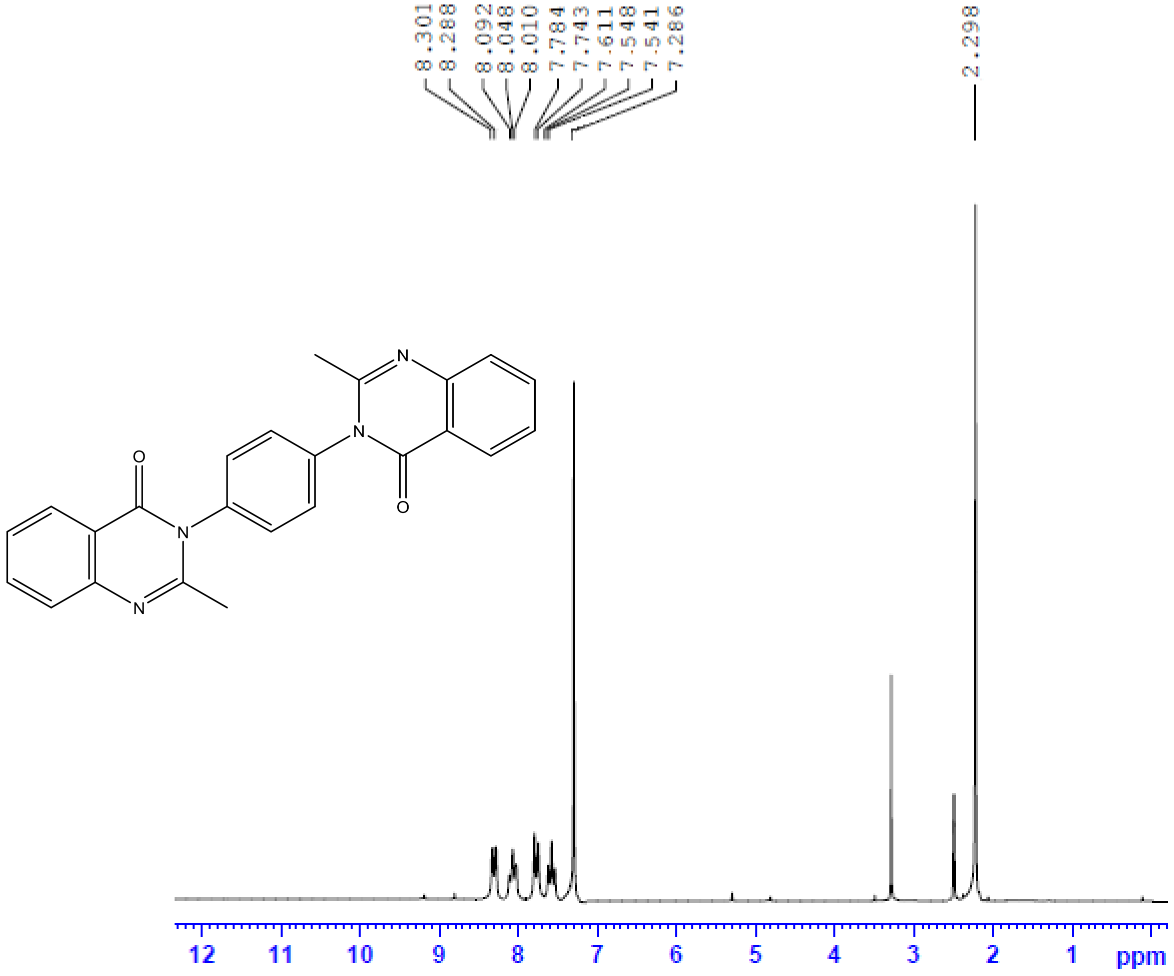
8.301
8.288
8.092
8.048
8.010
7.784
7.743
7.611
7.548
7.541
7.286
2.298
O
N
N
N
N
O
12
11
10
9
8
7
6
5
4
3
2
1
ppm

^{1}H NMR of **6a**

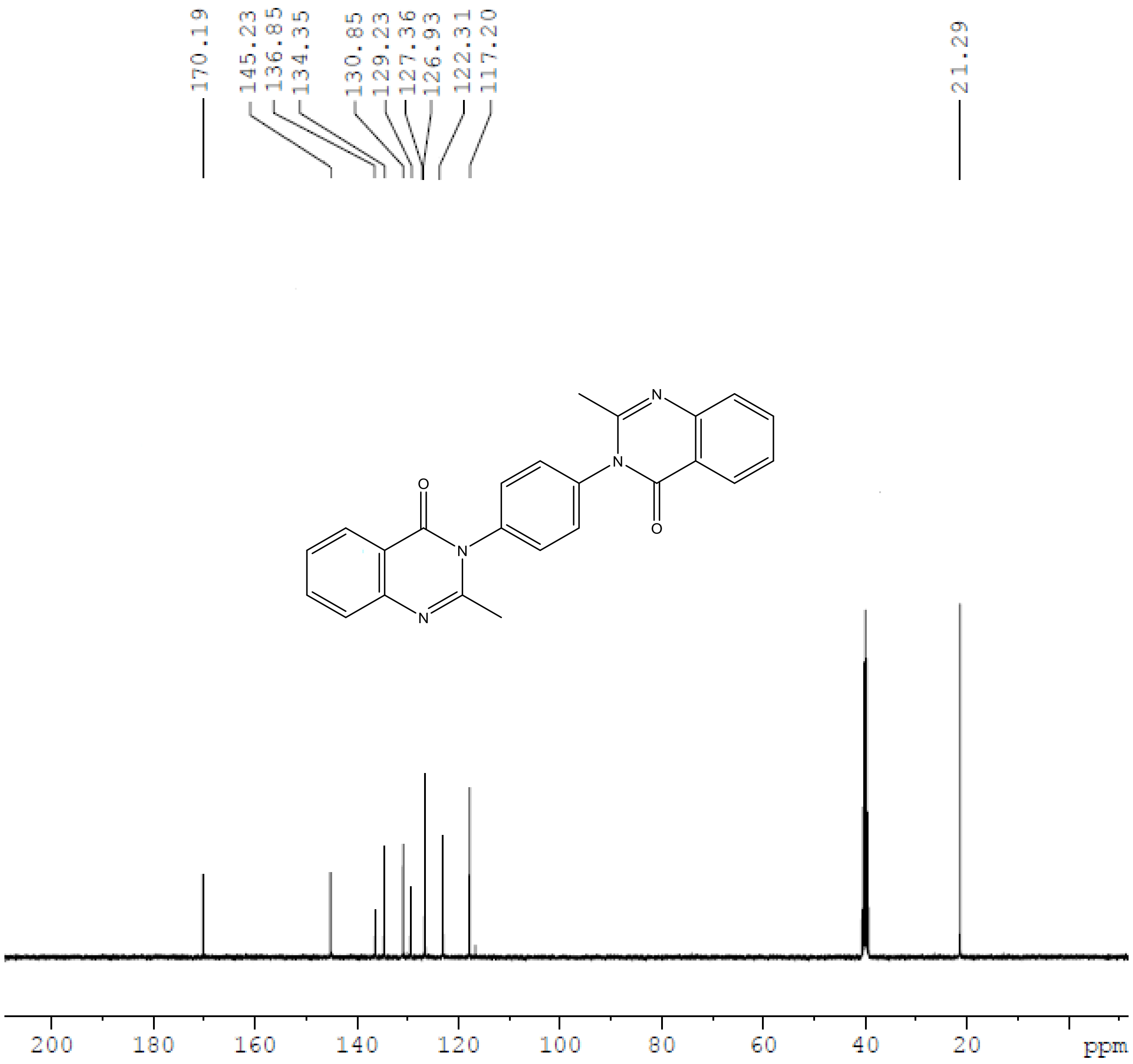
170.19
145.23
136.85
134.35
130.85
129.23
127.36
126.93
122.31
117.20
21.29
200
180
160
140
120
100
80
60
40
20
ppm

^{13}C NMR of **6a**

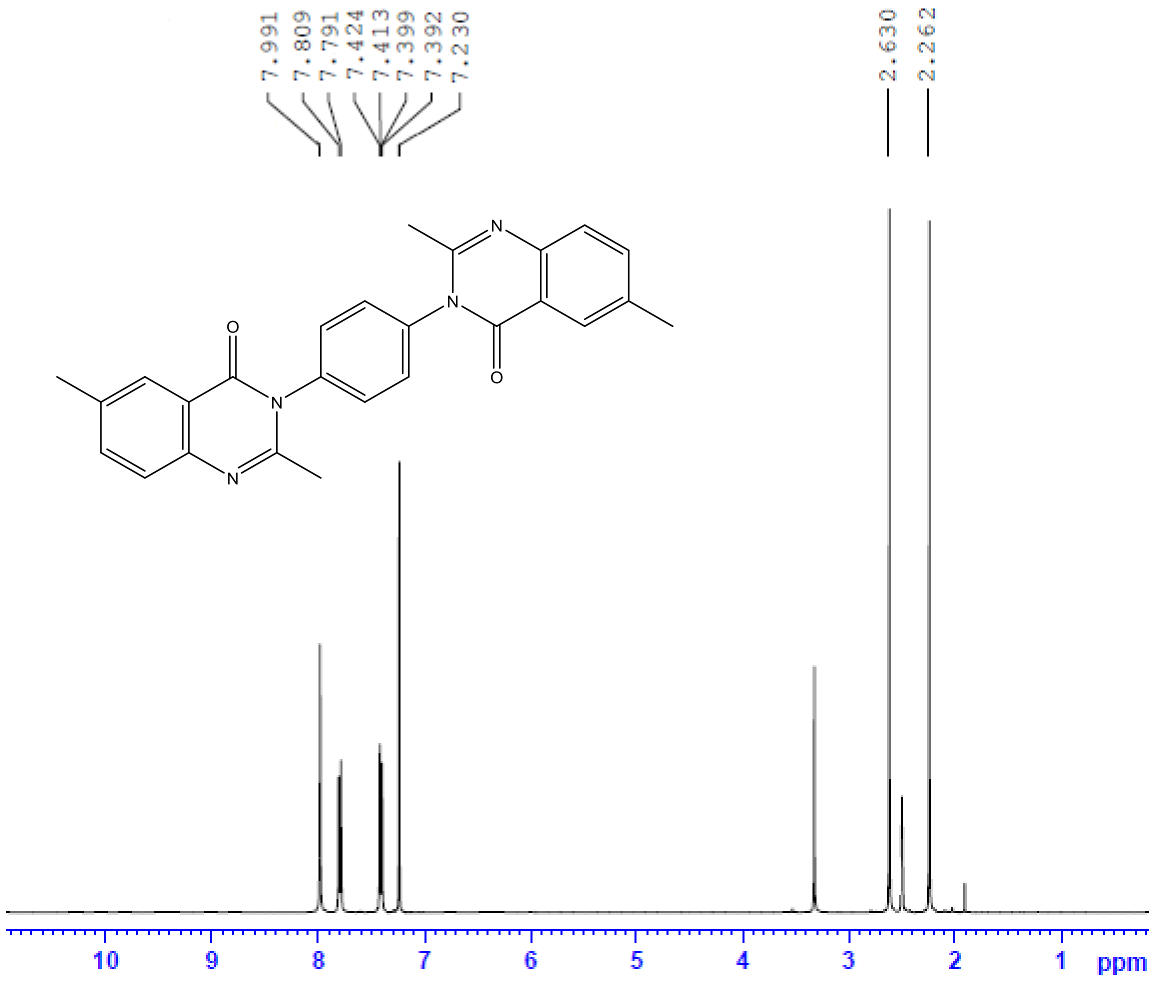
7.991
7.809
7.791
7.424
7.413
7.399
7.392
7.230
2.630
2.262
O
N
N
O
N
N
10
9
8
7
6
5
4
3
2
1
ppm

^{1}H NMR of **6b**

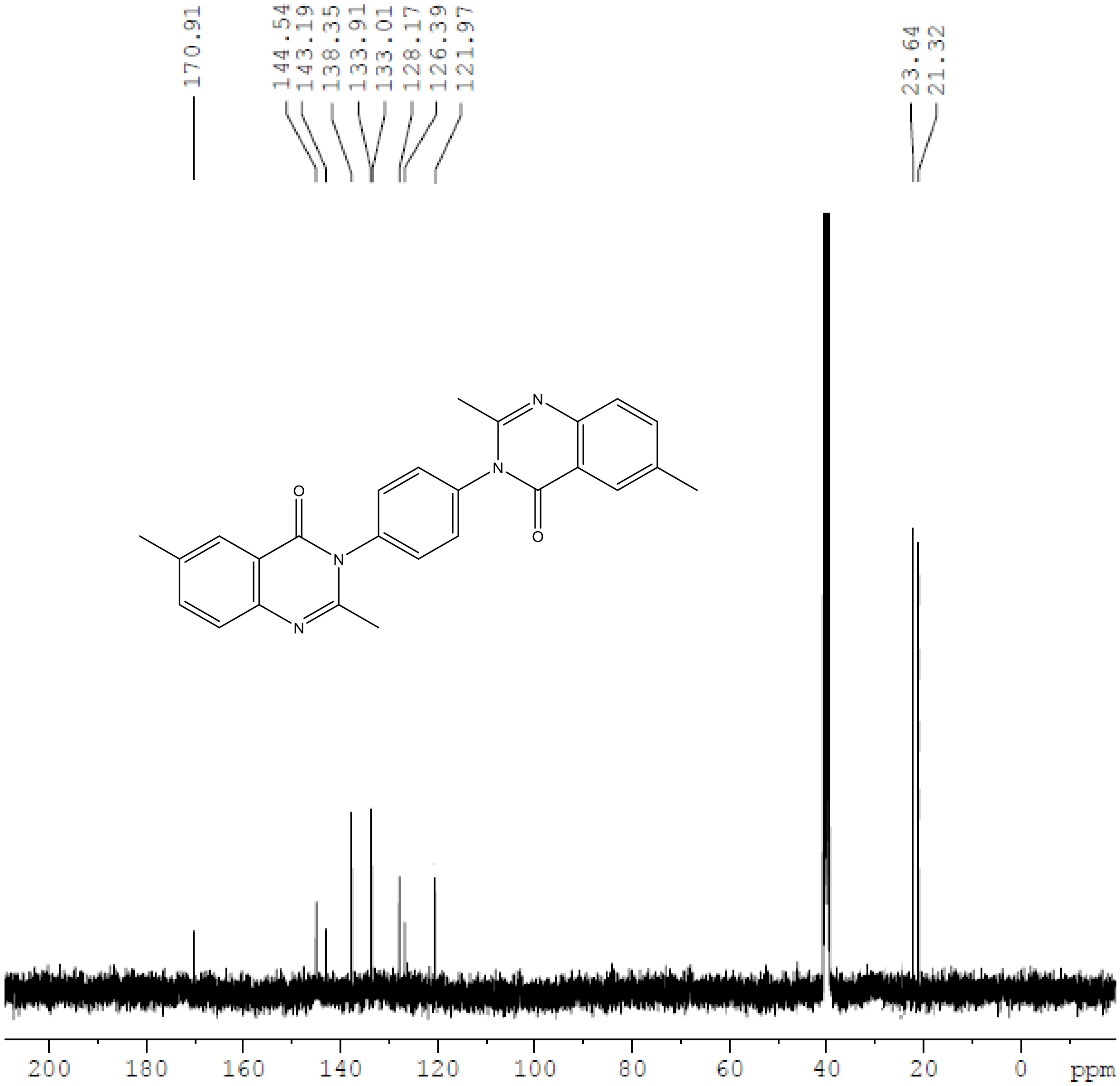
170.91
144.54
143.19
138.35
133.91
133.01
128.17
126.39
121.97
23.64
21.32
O
N
N
N
N
O
200
180
160
140
120
100
80
60
40
20
0
ppm

^{13}C NMR of **6b**

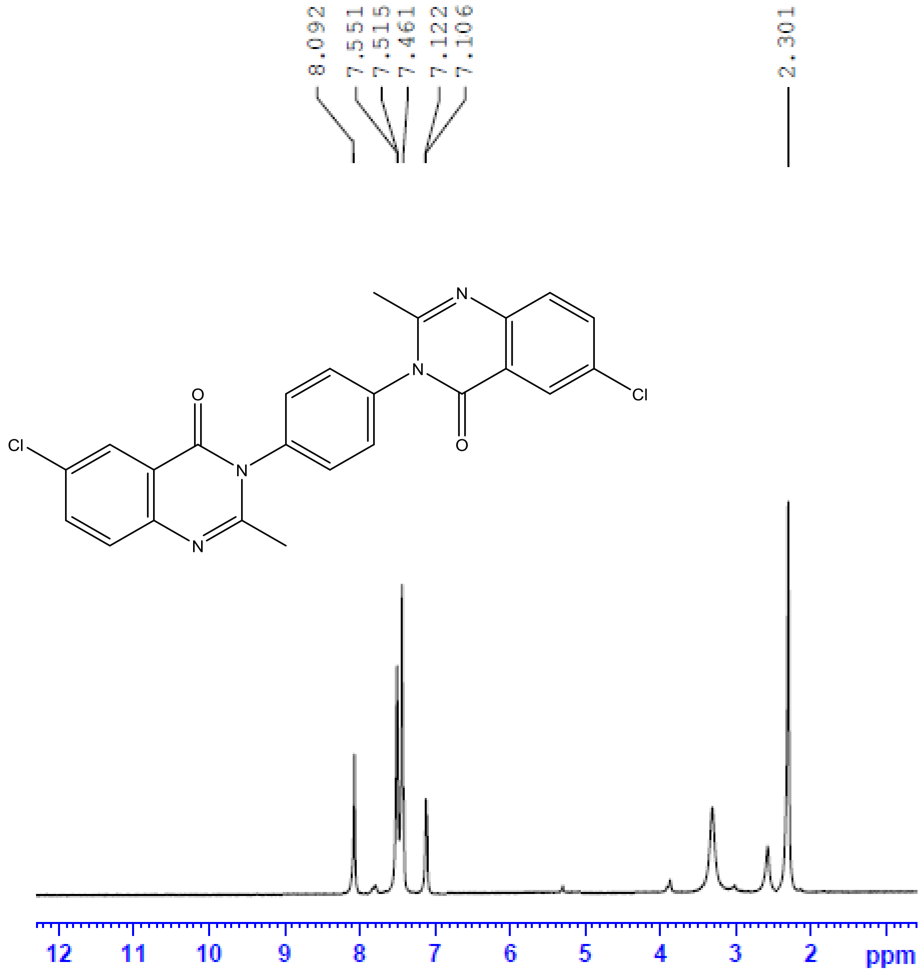
8.092
7.551
7.515
7.461
7.122
7.106
2.301
N
N
O
O
Cl
Cl
N
N
12
11
10
9
8
7
6
5
4
3
2
ppm

^{1}H NMR of **6c**

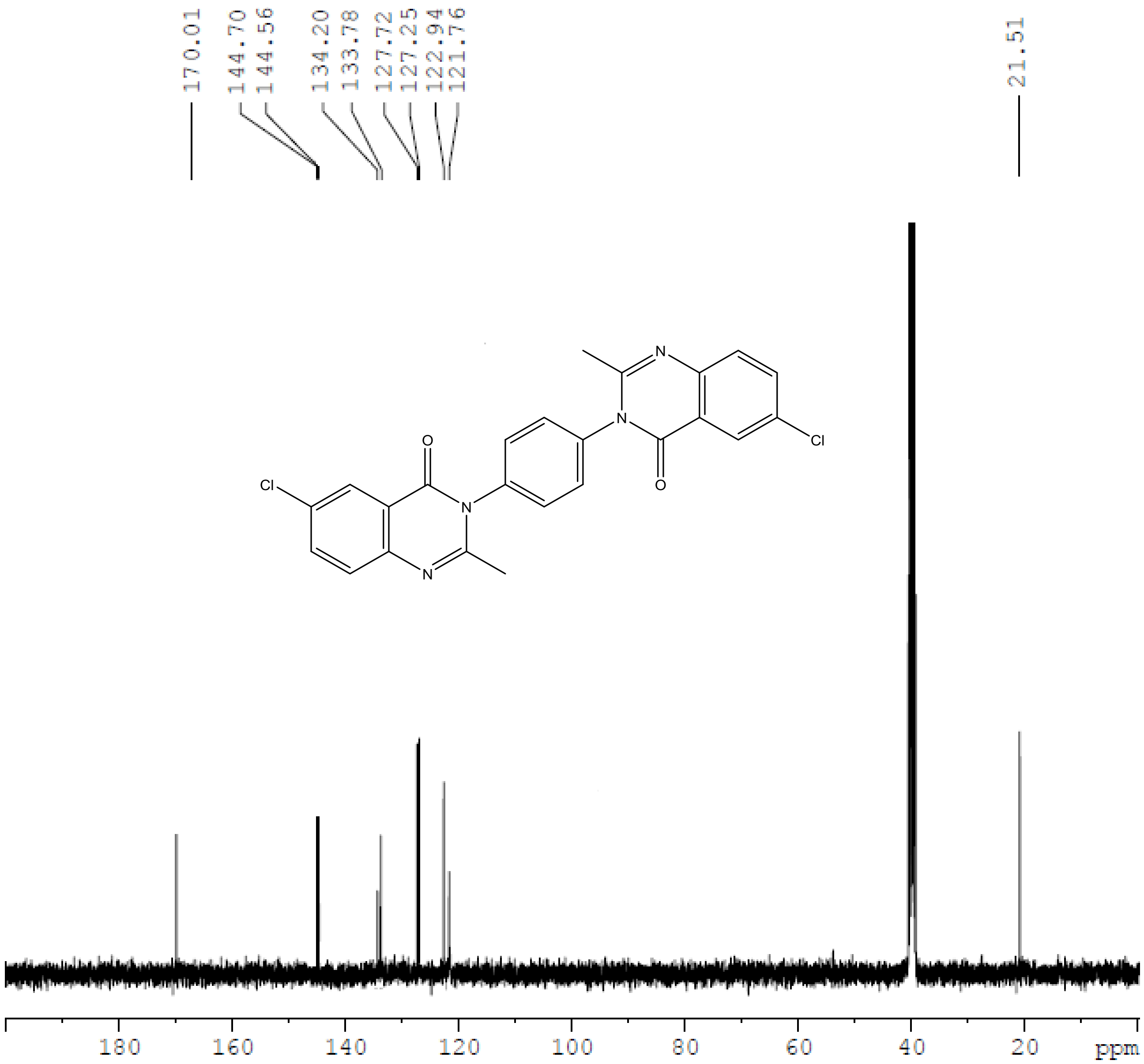
170.01
144.70
144.56
134.20
133.78
127.72
127.25
122.94
121.76
21.51
Cl
O
N
N
N
N
O
Cl
180
160
140
120
100
80
60
40
20
ppm

^{13}C NMR of **6c**

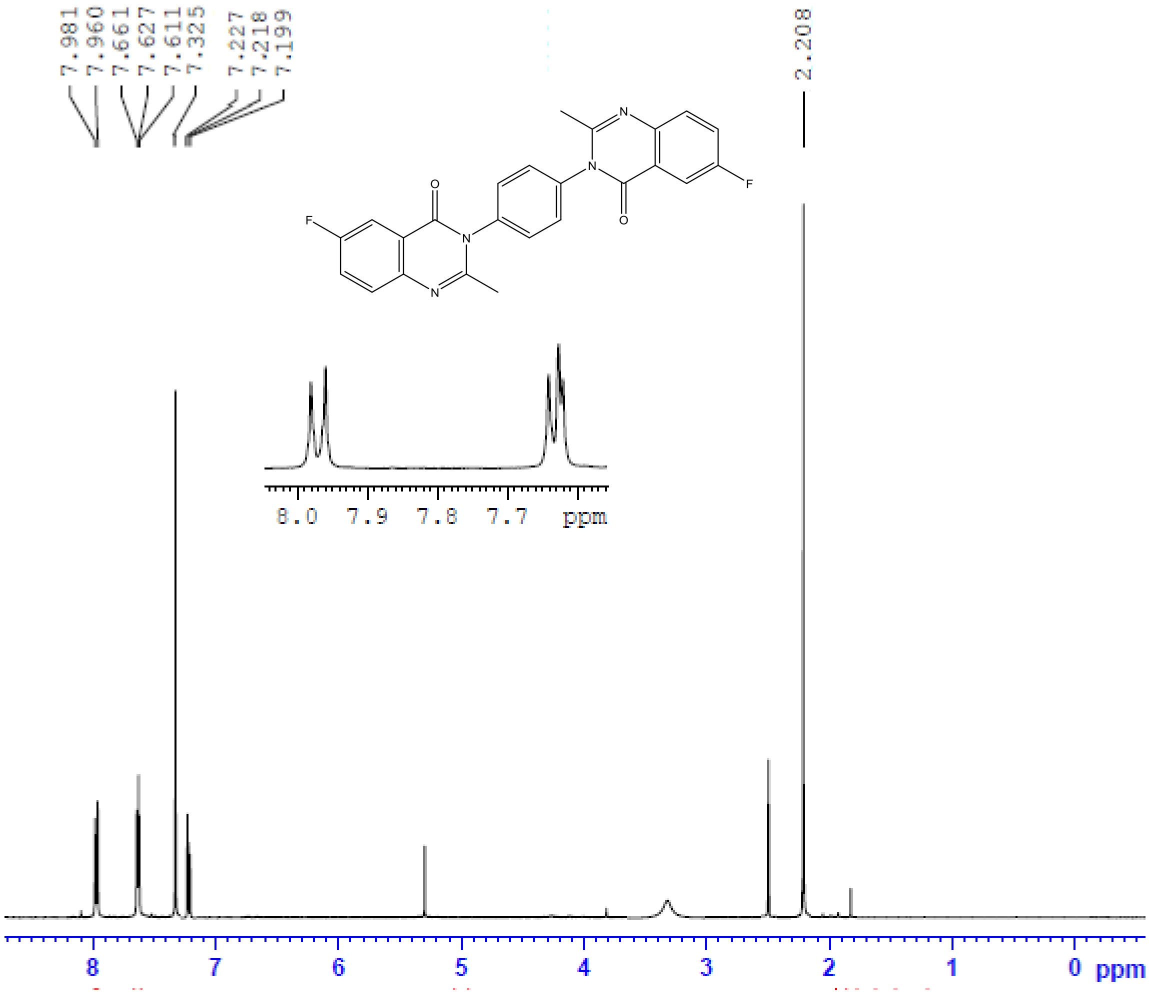
7.981
7.960
7.661
7.627
7.611
7.325
7.227
7.218
7.199
2.208
F
O
N
N
F
O
N
N
8.0 7.9 7.8 7.7 ppm
8 7 6 5 4 3 2 1 0 ppm

^{1}H NMR of **6d**

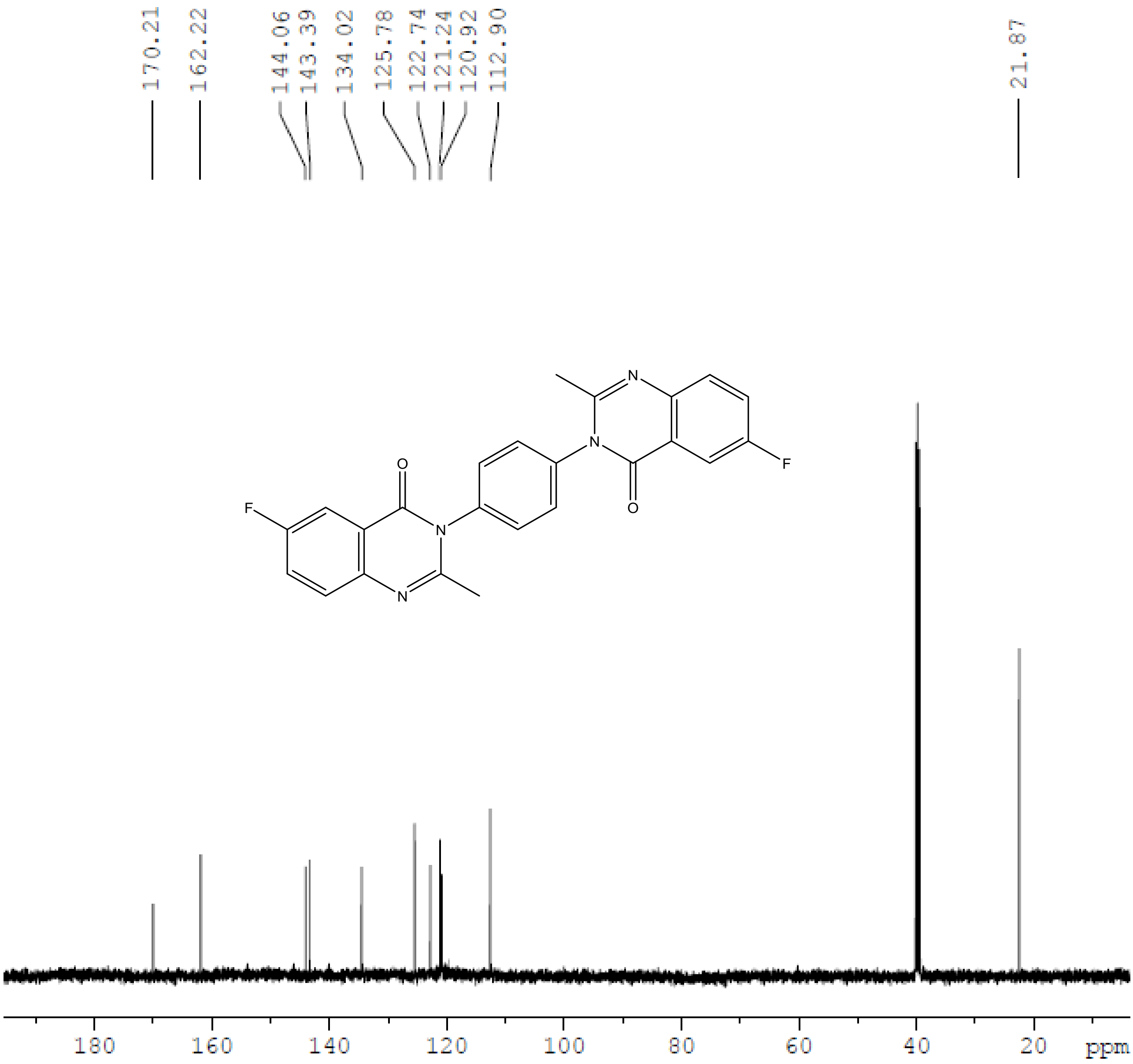
170.21
162.22
144.06
143.39
134.02
125.78
122.74
121.24
120.92
112.90
21.87
180
160
140
120
100
80
60
40
20
ppm

^{13}C NMR of **6d**

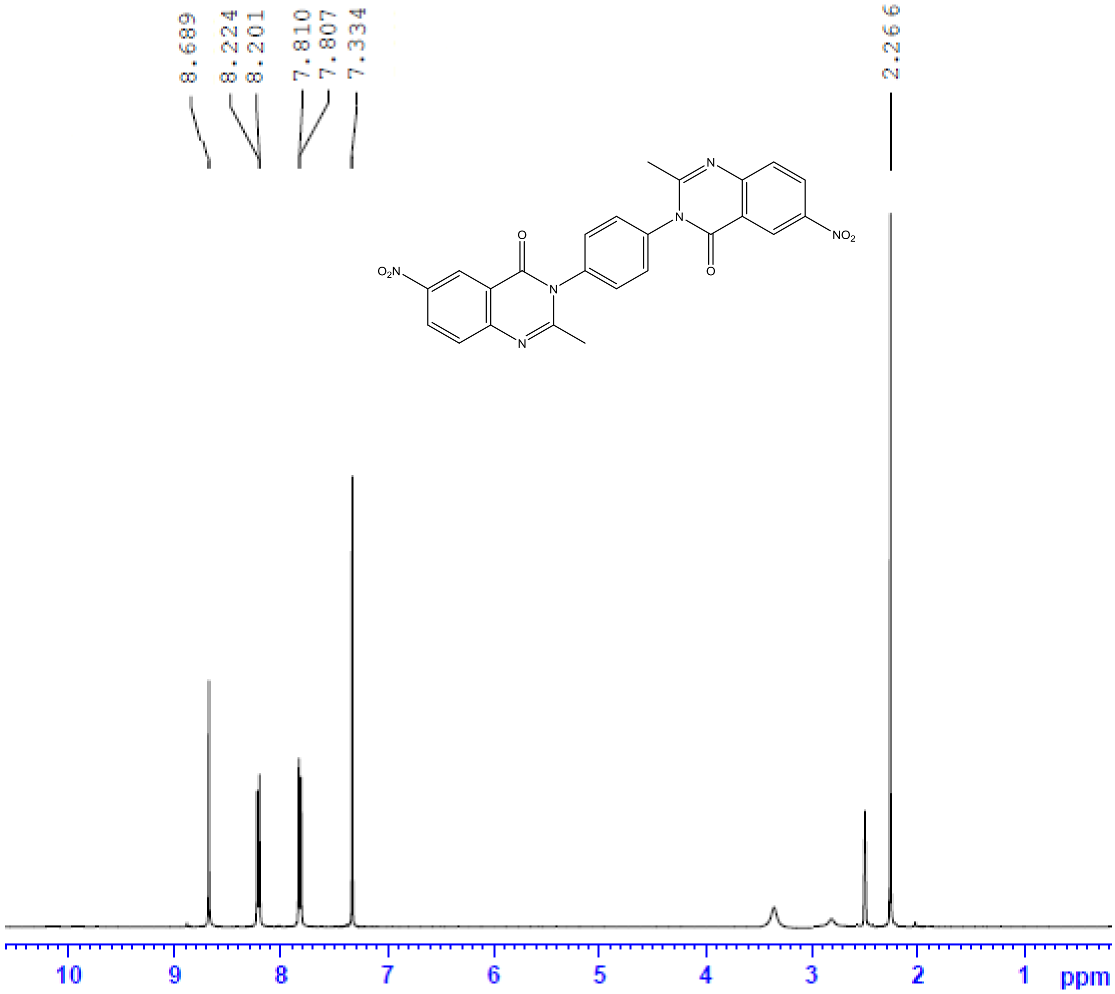
8.689
8.224
8.201
7.810
7.807
7.334
2.266
O
O₂N
N
N
O
N
N
NO₂
O
10
9
8
7
6
5
4
3
2
1
ppm

^{1}H NMR of **6e**

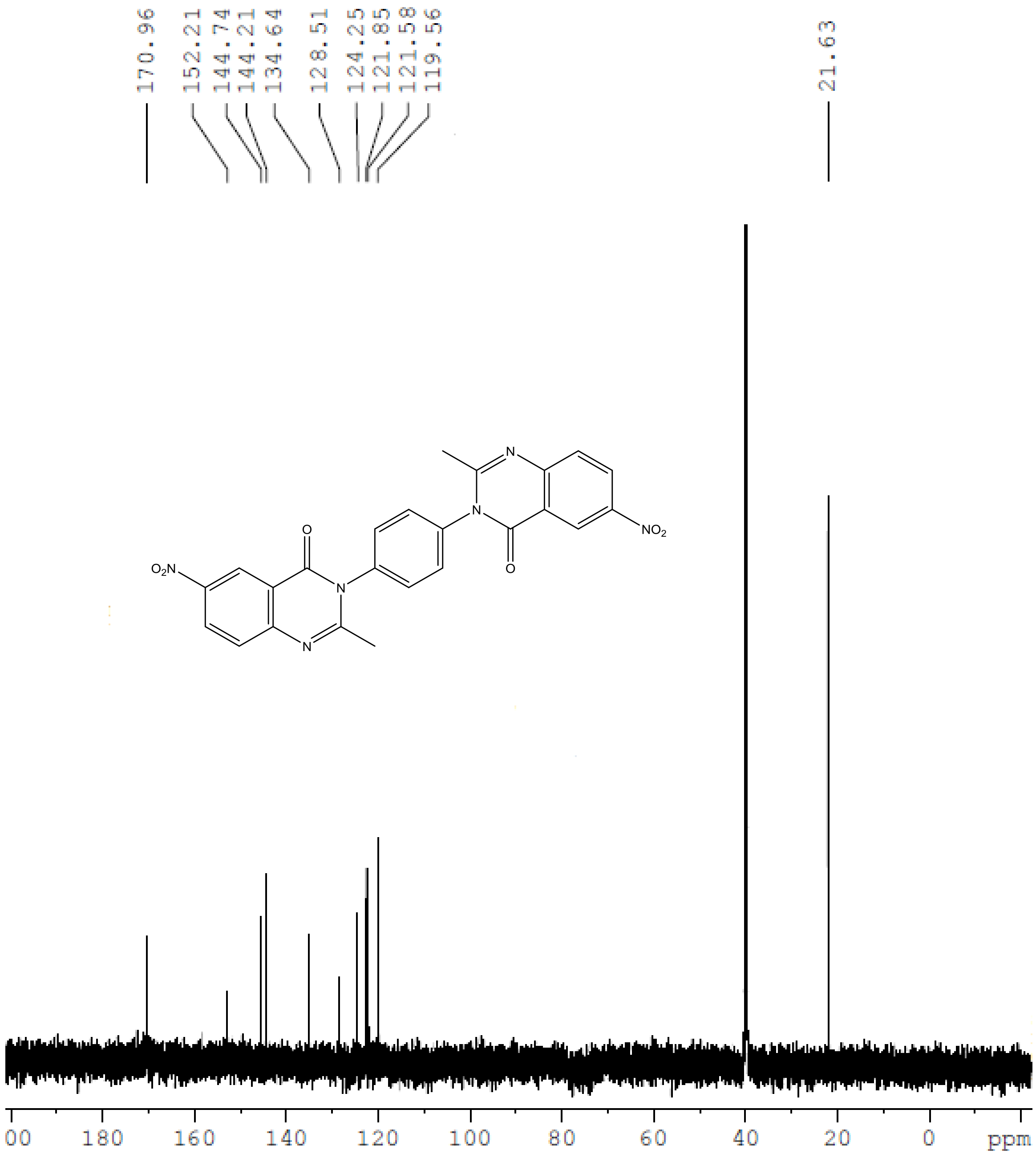
170.96
152.21
144.74
144.21
134.64
128.51
124.25
121.85
121.58
119.56
21.63
O
O2N
N
N
N
N
O
NO2
00
180
160
140
120
100
80
60
40
20
0
ppm

^{13}C NMR of **6e**

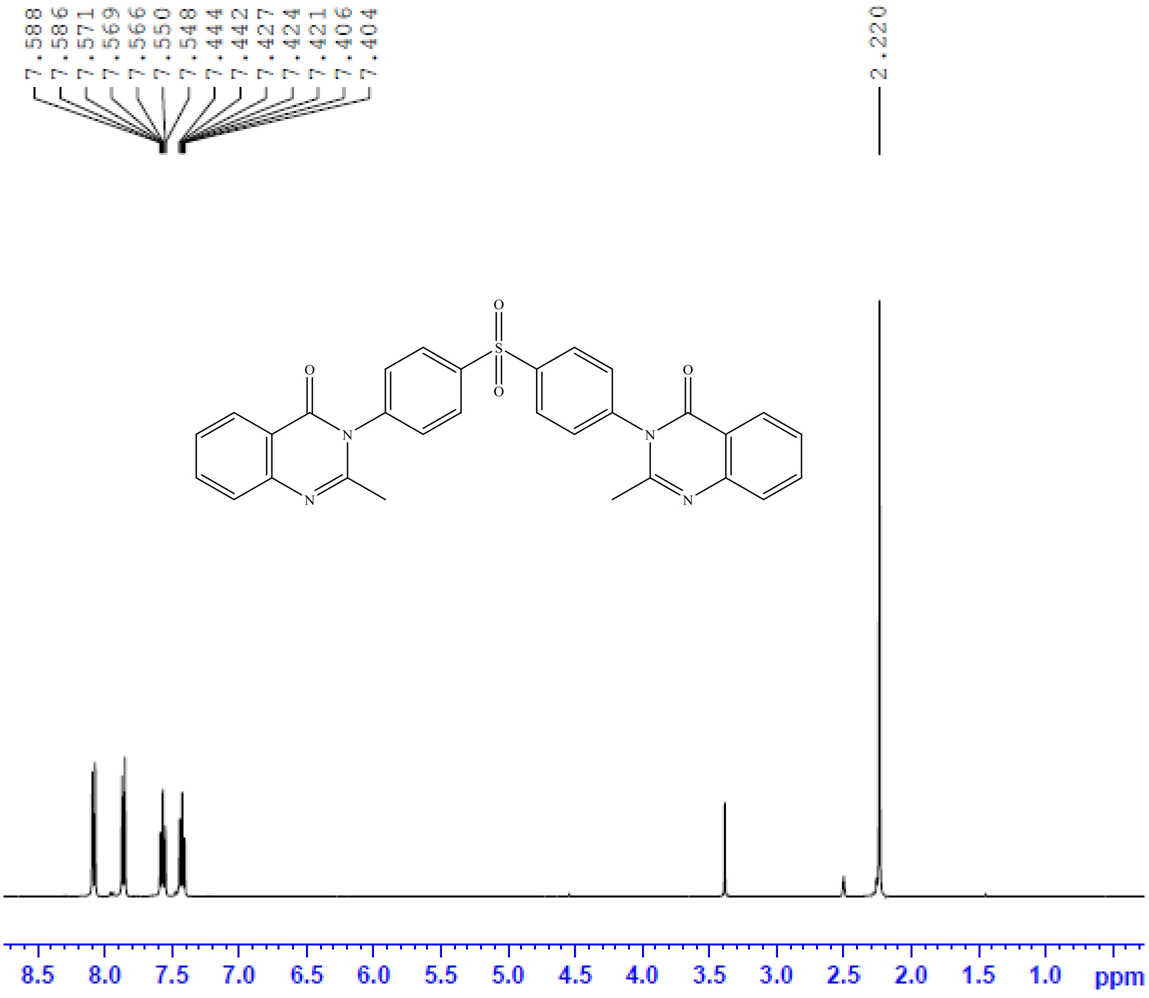
7.588
7.586
7.571
7.569
7.566
7.550
7.548
7.444
7.442
7.427
7.424
7.421
7.406
7.404
2.220
O
S
O
O
N
N
O
N
N
8.5 8.0 7.5 7.0 6.5 6.0 5.5 5.0 4.5 4.0 3.5 3.0 2.5 2.0 1.5 1.0 ppm

^{1}H NMR of **7a**

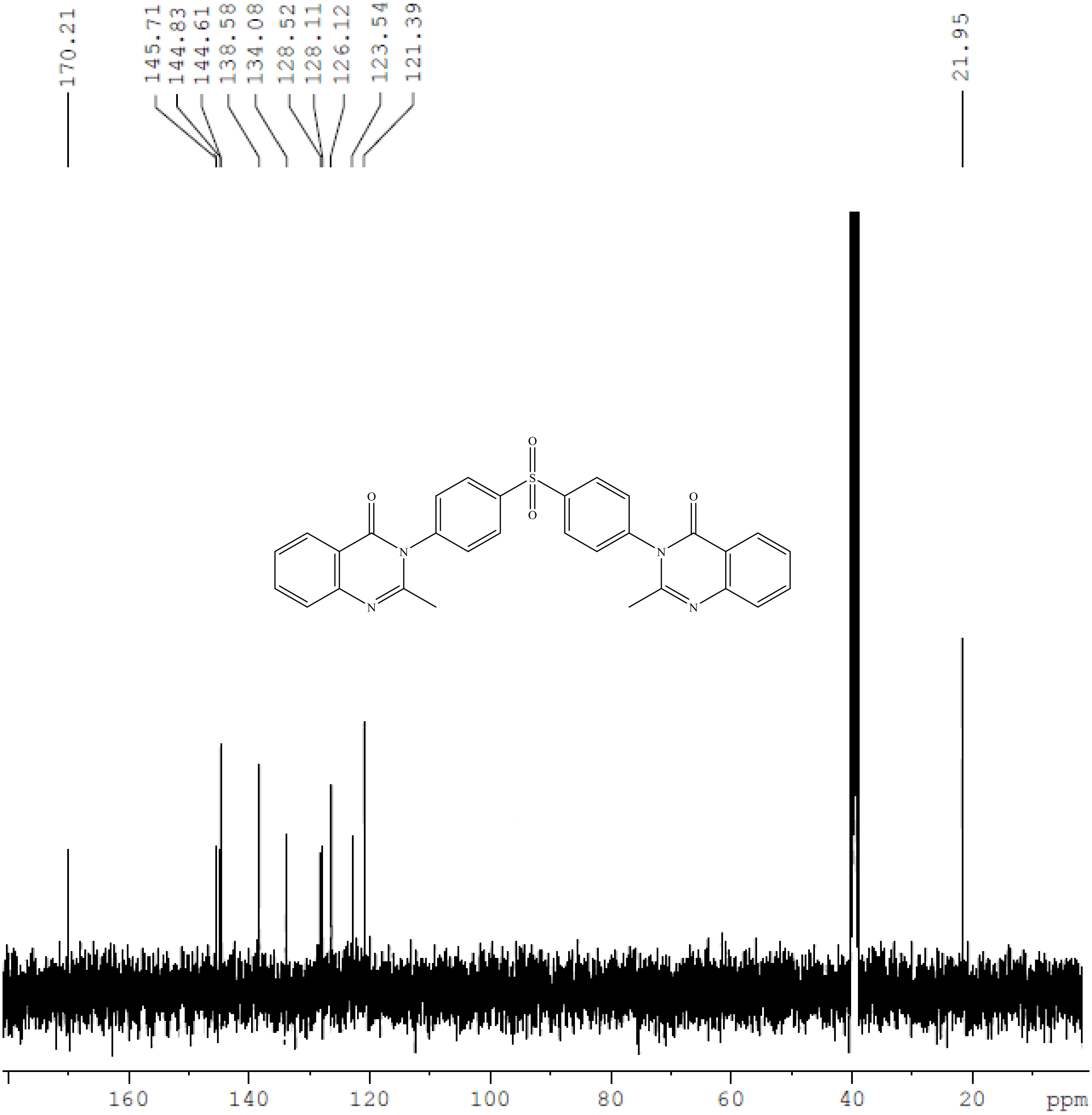
170.21
145.71
144.83
144.61
138.58
134.08
128.52
128.11
126.12
123.54
121.39
21.95
O
S
O
O
N
N
O
N
N
160
140
120
100
80
60
40
20
ppm

^{13}C NMR of **7a**

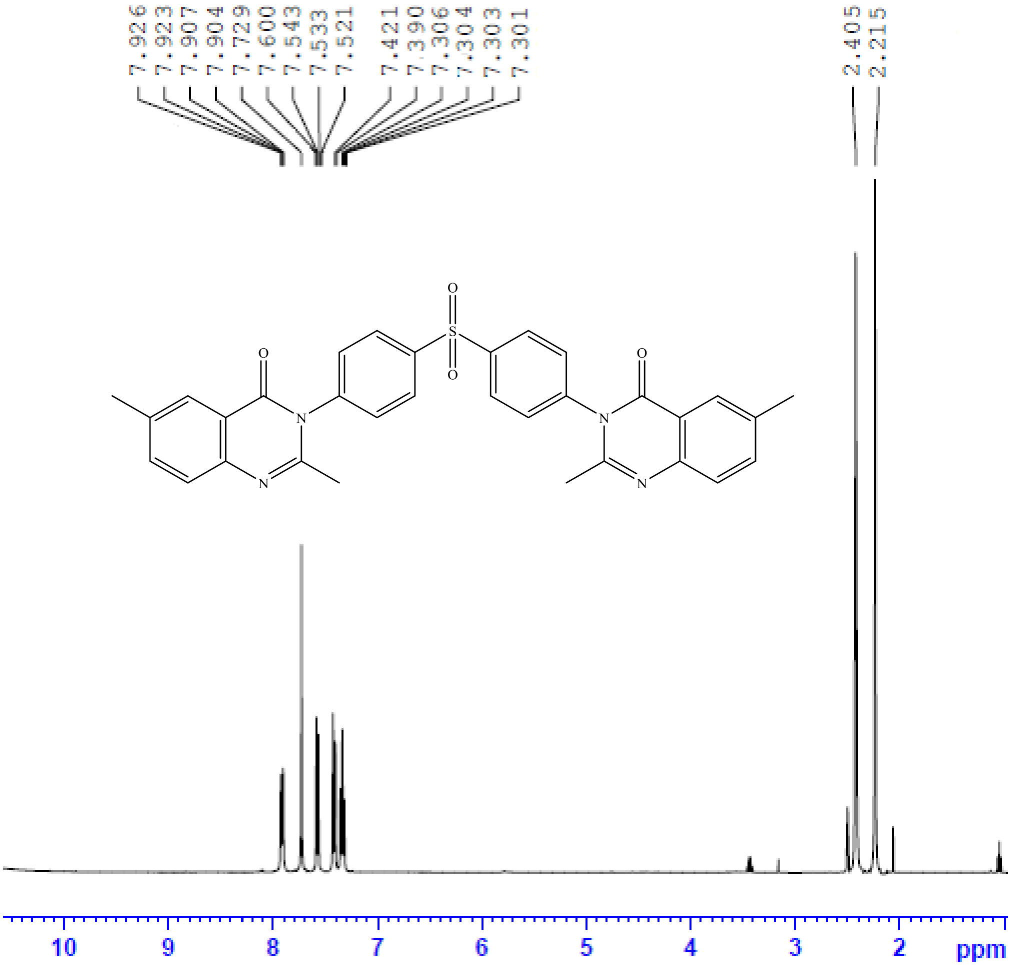
7.926
7.923
7.907
7.904
7.729
7.600
7.543
7.533
7.521
7.421
7.390
7.306
7.304
7.303
7.301
2.405
2.215
O
S
O
O
N
N
O
N
N
10
9
8
7
6
5
4
3
2
ppm

^{1}H NMR of **7b**

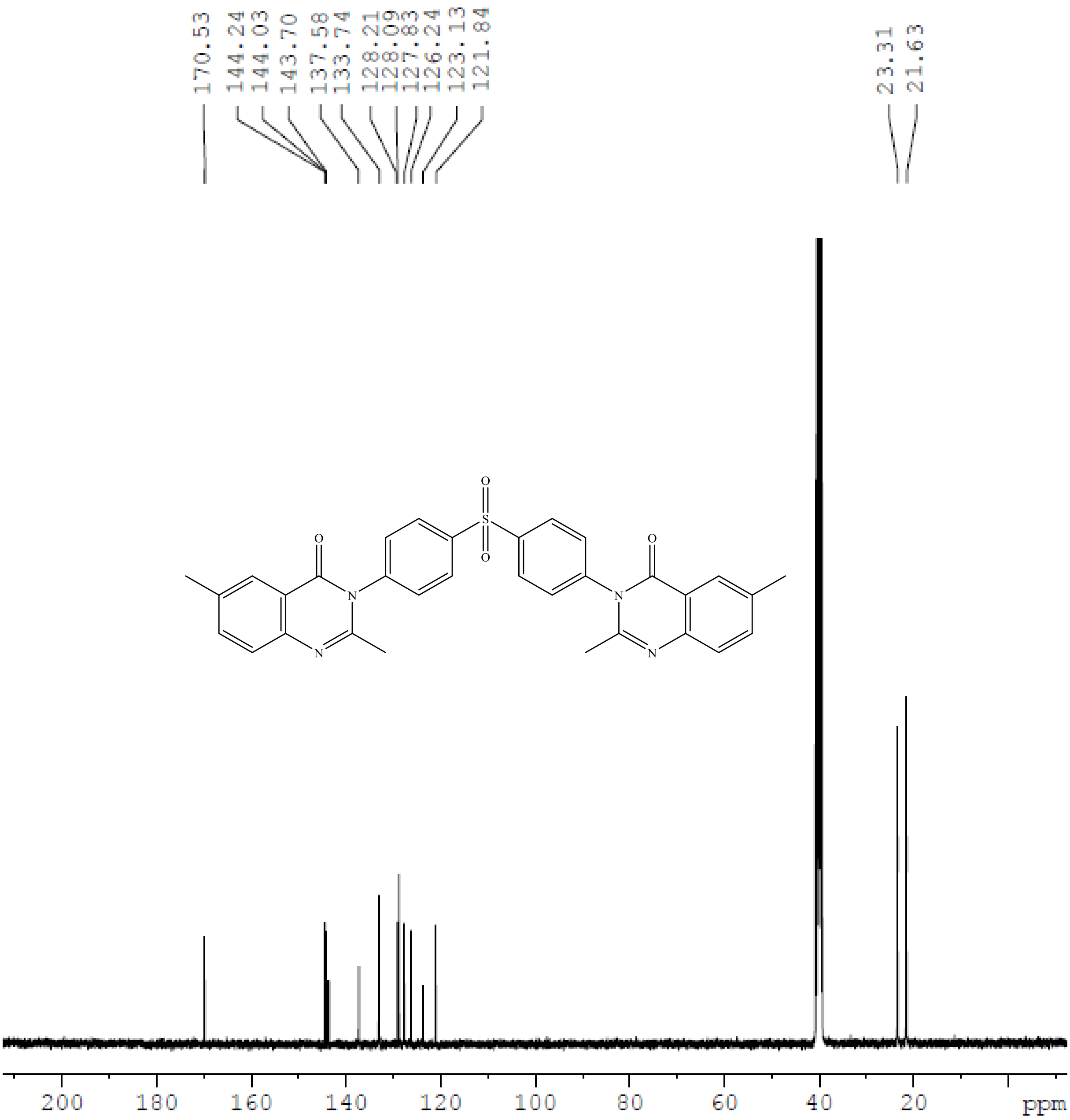
170.53
144.24
144.03
143.70
137.58
133.74
128.21
128.09
127.83
126.24
123.13
121.84
23.31
21.63
200
180
160
140
120
100
80
60
40
20
ppm

^{13}C NMR of **7b**

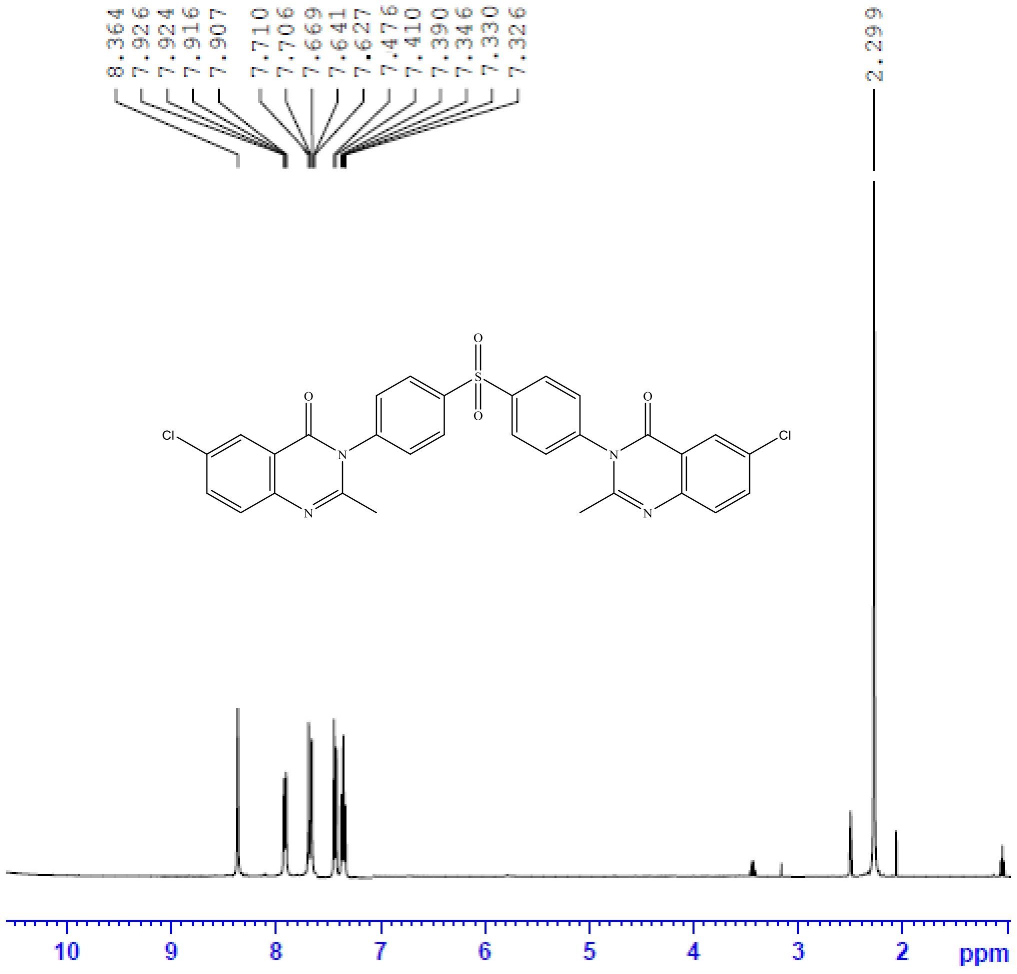
8.364
7.926
7.924
7.916
7.907
7.710
7.706
7.669
7.641
7.627
7.476
7.410
7.390
7.346
7.330
7.326
2.299
O
S
O
O
O
Cl
N
N
N
N
Cl
10
9
8
7
6
5
4
3
2
ppm

^{1}H NMR of **7c**

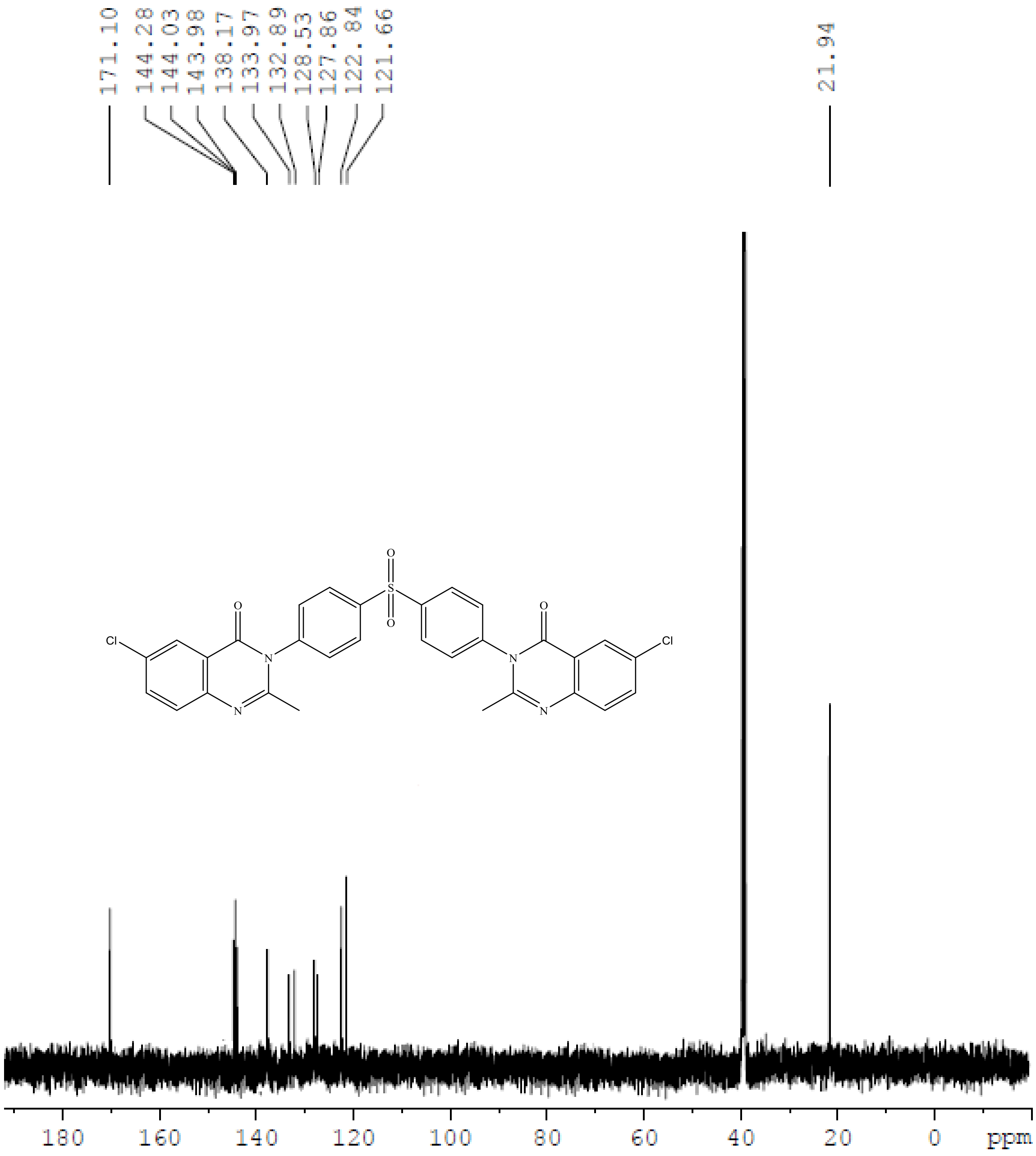

171.10
144.28
144.03
143.98
138.17
133.97
132.89
128.53
127.86
122.84
121.66
21.94
O
O
S
O
O
O
Cl
N
N
N
N
Cl
180
160
140
120
100
80
60
40
20
0
ppm

^{13}C NMR of **7c**

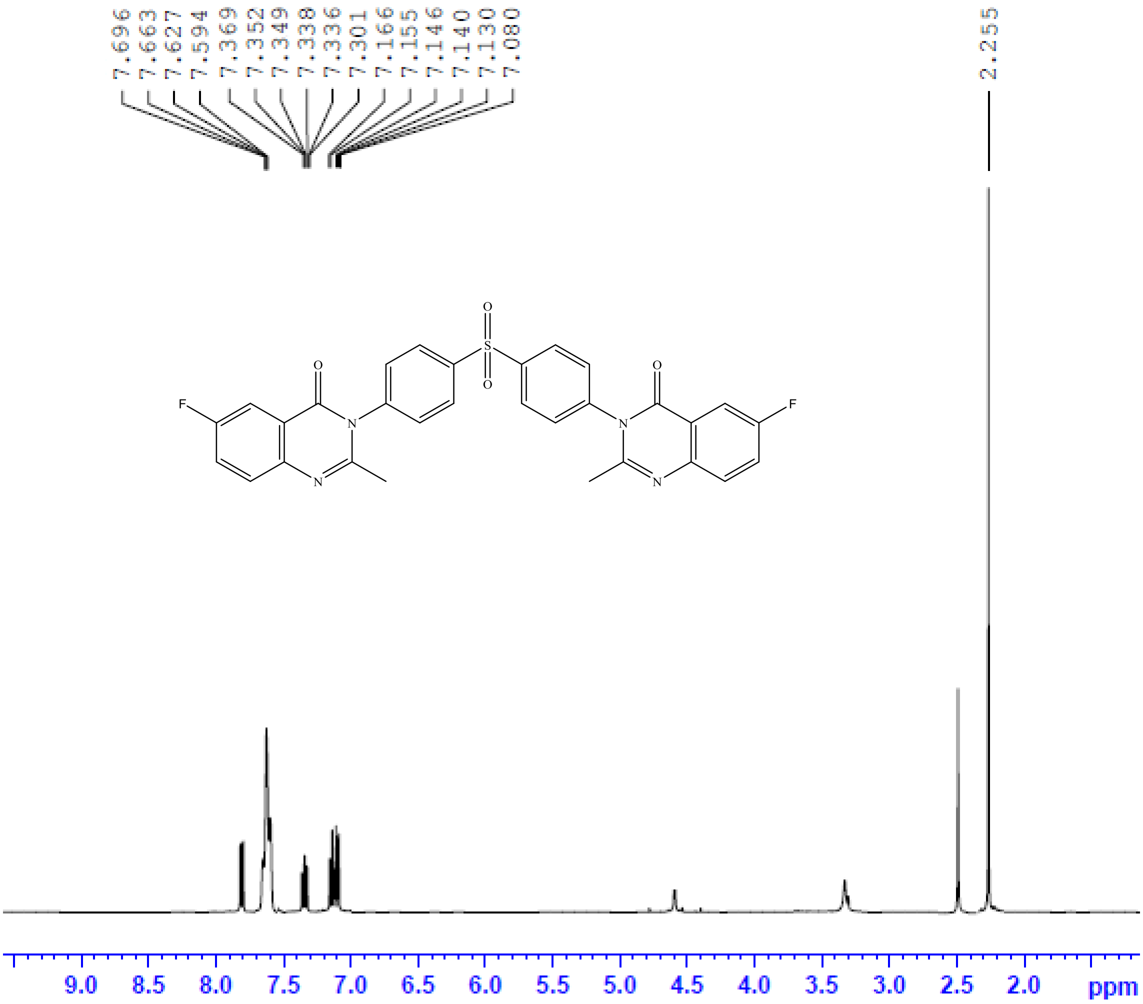
7.696
7.663
7.627
7.594
7.369
7.352
7.349
7.338
7.336
7.301
7.166
7.155
7.146
7.140
7.130
7.080
2.255
O
S
O
O
N
F
N
O
N
F
N
9.0
8.5
8.0
7.5
7.0
6.5
6.0
5.5
5.0
4.5
4.0
3.5
3.0
2.5
2.0
ppm

^{1}H NMR of **7d**

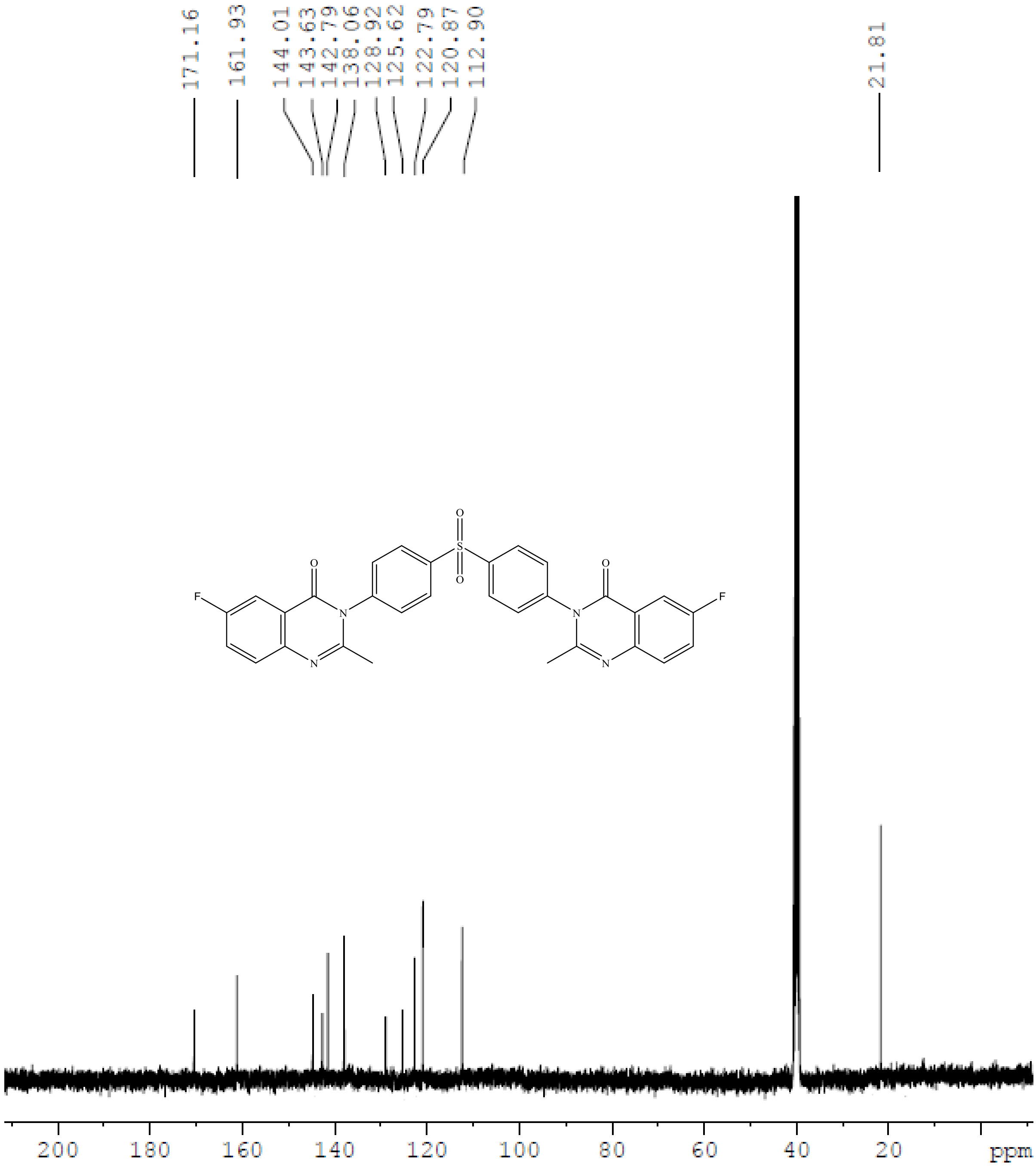

171.16
161.93
144.01
143.63
142.79
138.06
128.92
125.62
122.79
120.87
112.90
21.81
F
O
N
N
S
O
O
O
N
N
F
200
180
160
140
120
100
80
60
40
20
ppm

^{13}C NMR of **7d**

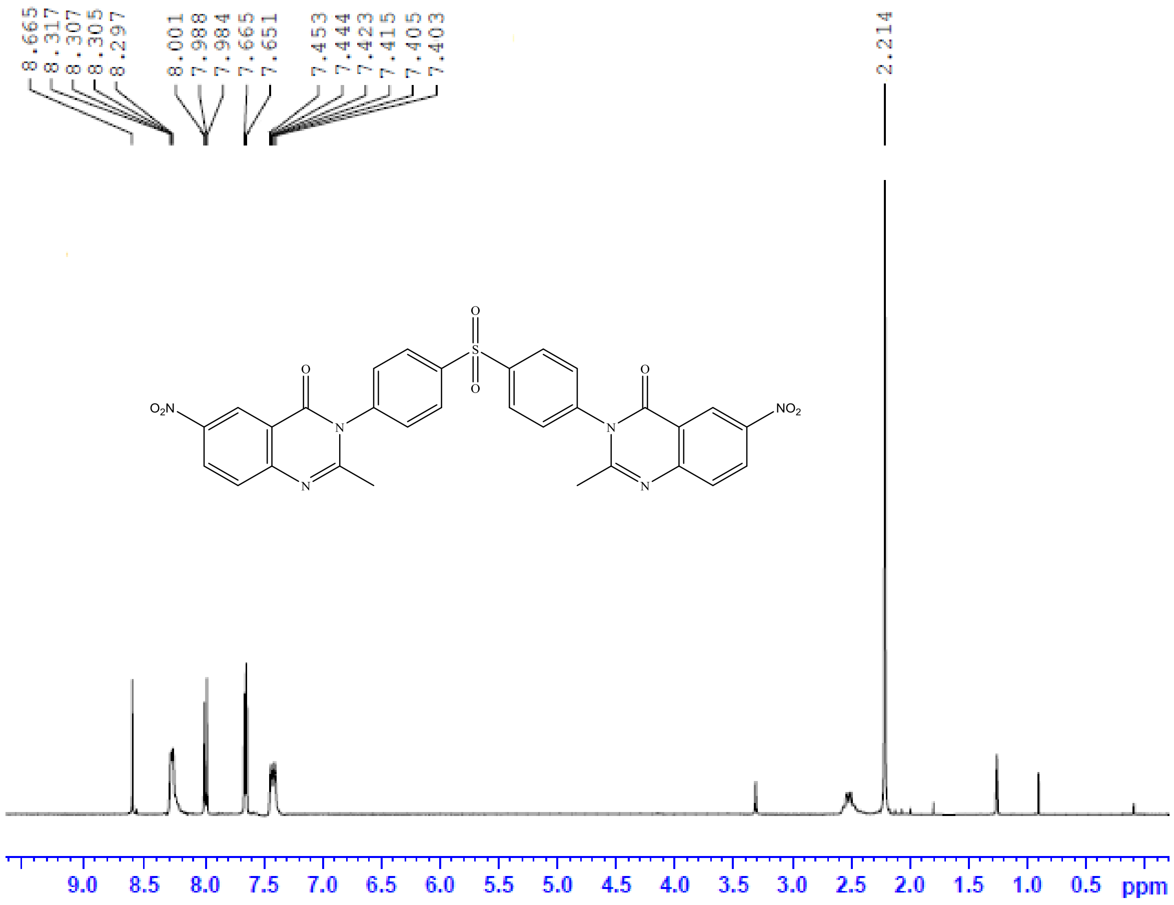
8.665
8.317
8.307
8.305
8.297
8.001
7.988
7.984
7.665
7.651
7.453
7.444
7.423
7.415
7.405
7.403
2.214
O₂N
NO₂
ppm

^{1}H NMR of **7e**

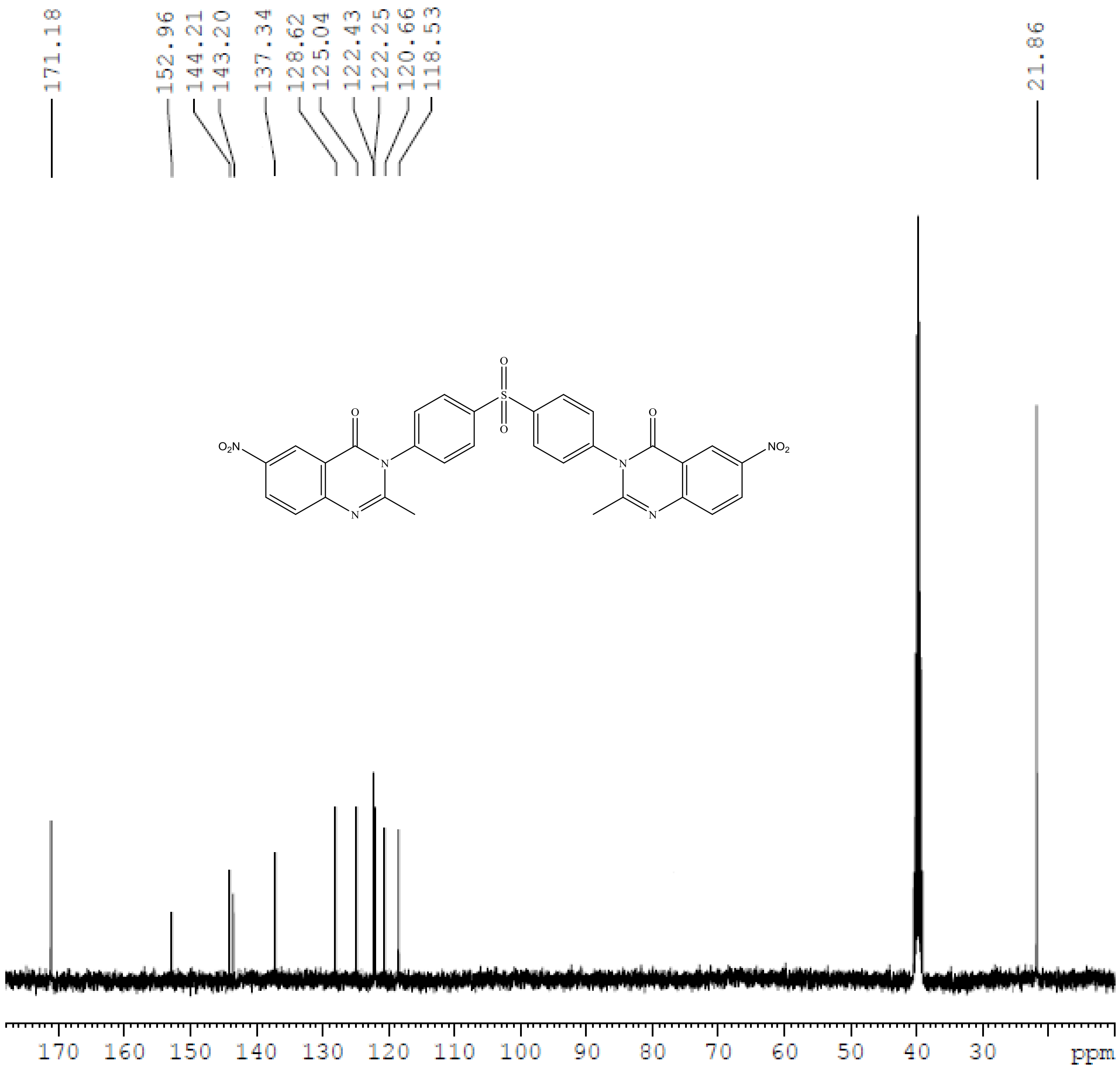

171.18
152.96
144.21
143.20
137.34
128.62
125.04
122.43
122.25
120.66
118.53
21.86
O
O
S
O
O
O_2N
N
N
O
N
N
NO_2
170 160 150 140 130 120 110 100 90 80 70 60 50 40 30 ppm

^{13}C NMR of **7e**

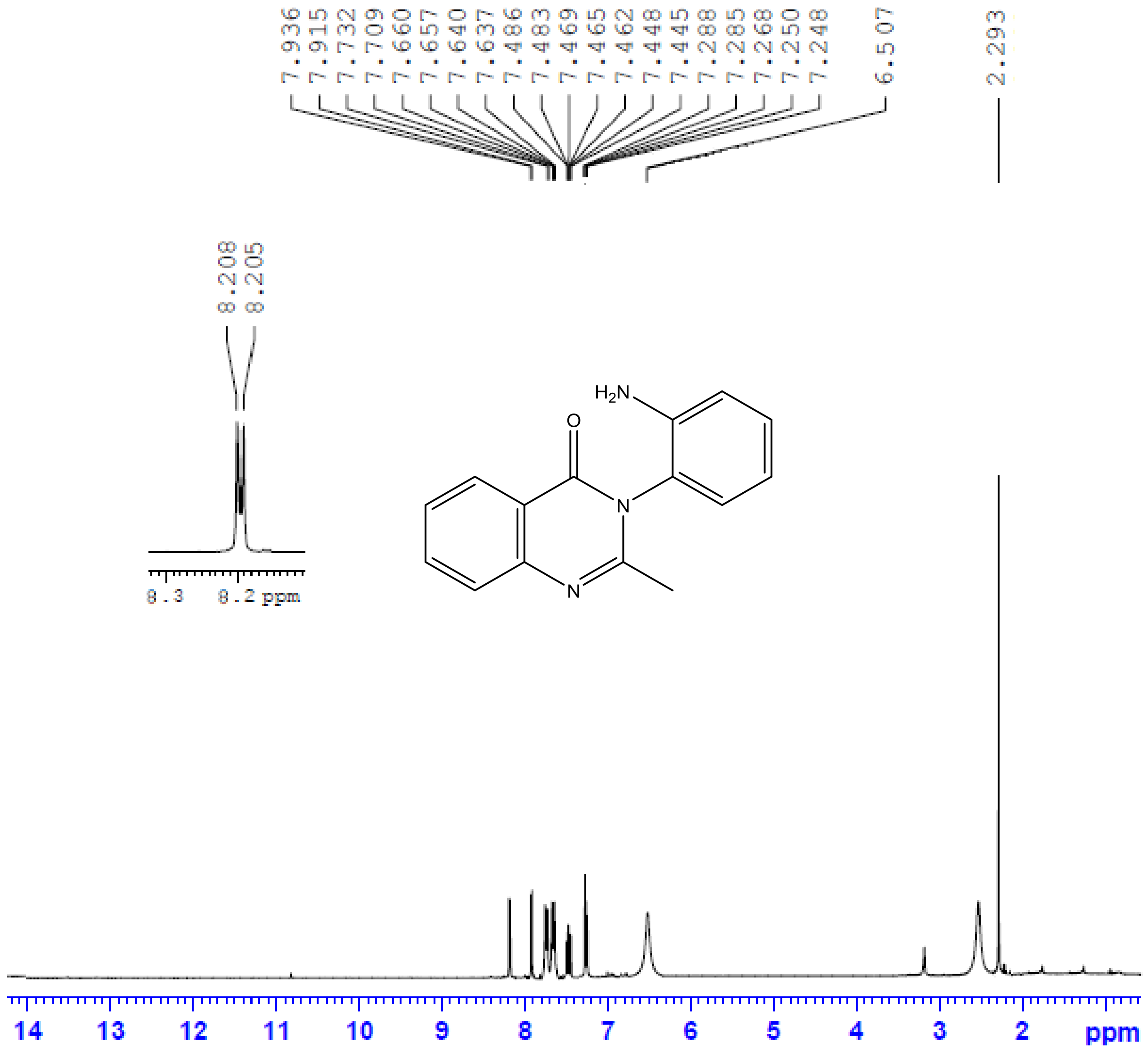
7.936
7.915
7.732
7.709
7.660
7.657
7.640
7.637
7.486
7.483
7.469
7.465
7.462
7.448
7.445
7.288
7.285
7.268
7.250
7.248
6.507
2.293
8.208
8.205
8.3
8.2 ppm
H2N
O
N
N
14
13
12
11
10
9
8
7
6
5
4
3
2
ppm

^{1}H NMR of **8**

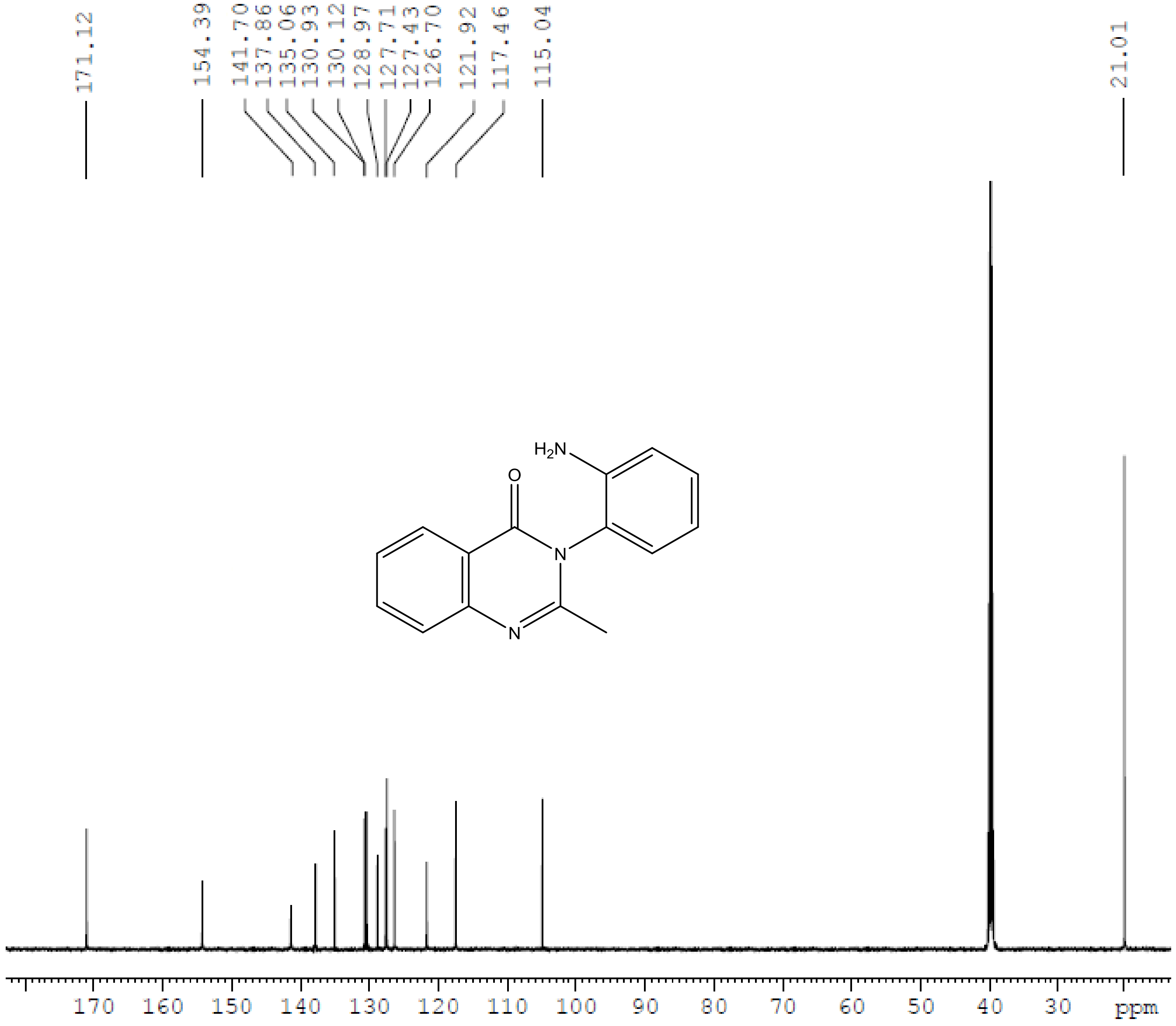

171.12
154.39
141.70
137.86
135.06
130.93
130.12
128.97
127.71
127.43
126.70
121.92
117.46
115.04
21.01
H2N
O
N
N
170 160 150 140 130 120 110 100 90 80 70 60 50 40 30 ppm

^{13}C NMR of **8**

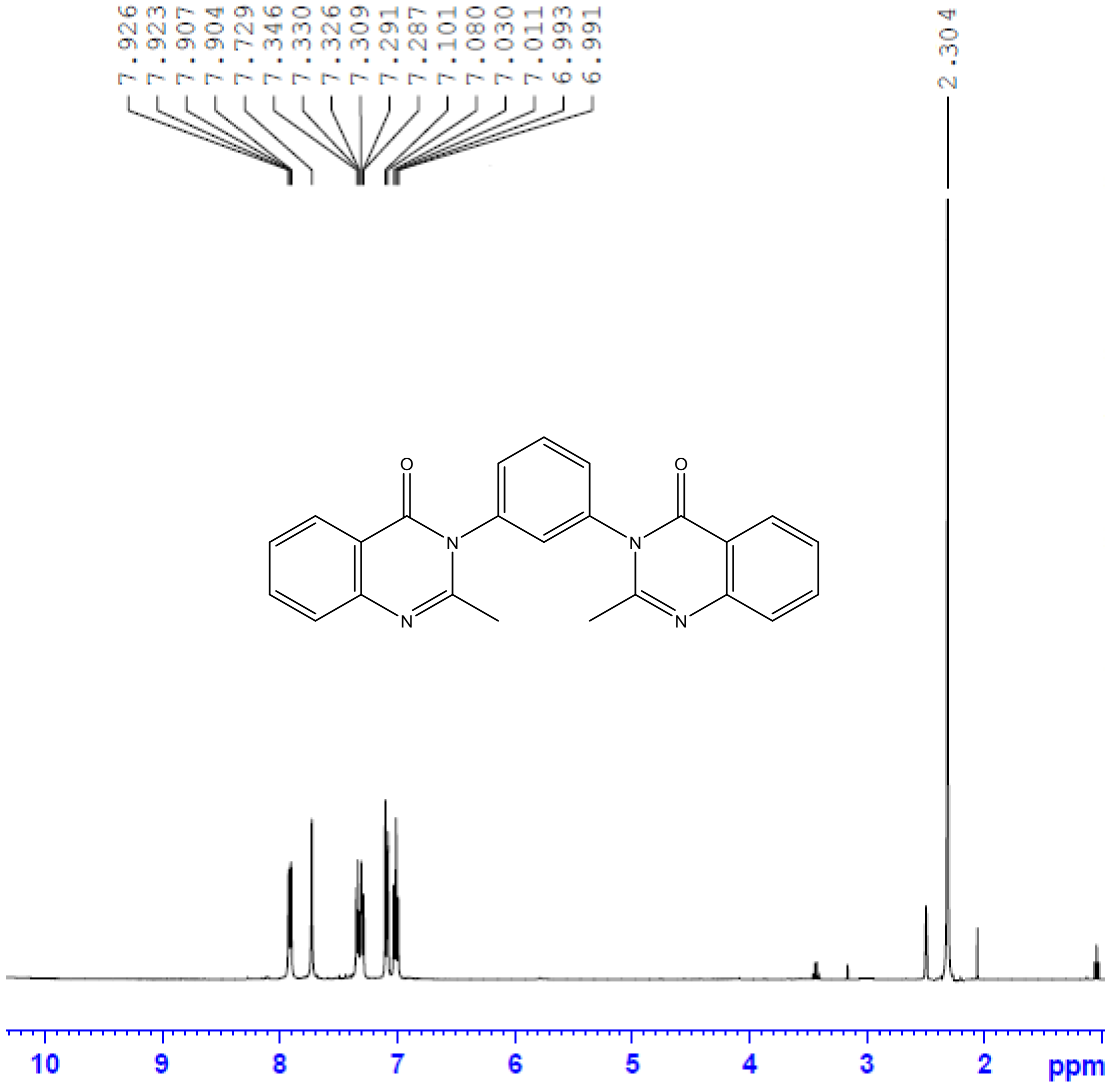

7.926
7.923
7.907
7.904
7.729
7.346
7.330
7.326
7.309
7.291
7.287
7.101
7.080
7.030
7.011
6.993
6.991
2.304
O
N
N
O
N
N
10
9
8
7
6
5
4
3
2
ppm

^{1}H NMR of **11**

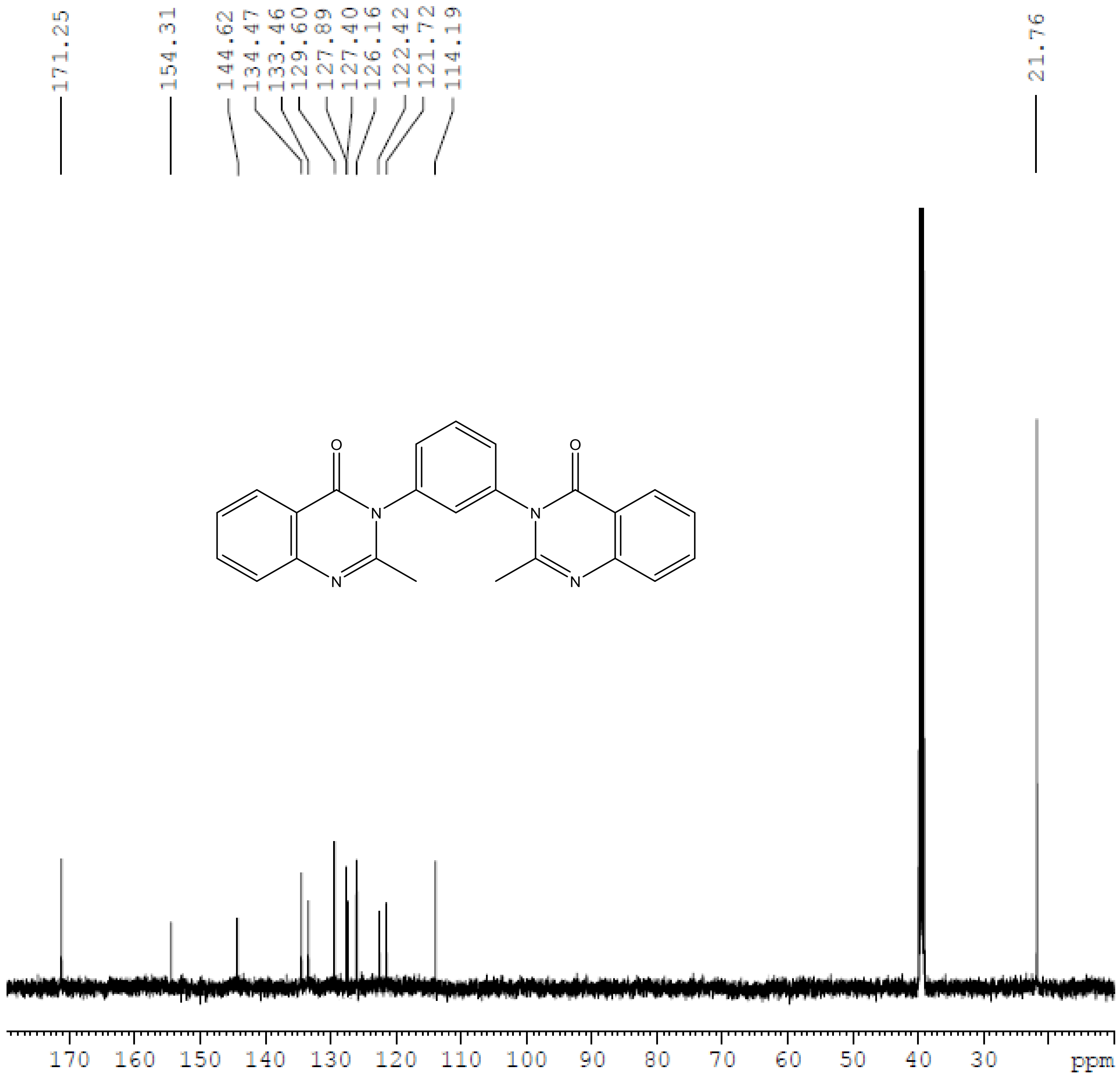

171.25
154.31
144.62
134.47
133.46
129.60
127.89
127.40
126.16
122.42
121.72
114.19
21.76
O
N
N
O
N
N
170
160
150
140
130
120
110
100
90
80
70
60
50
40
30
ppm

^{13}C NMR of **11**

Table 3. The anti-proliferative activities of the newly prepared bis-quinazolinone derivatives against human cancer cell lines.

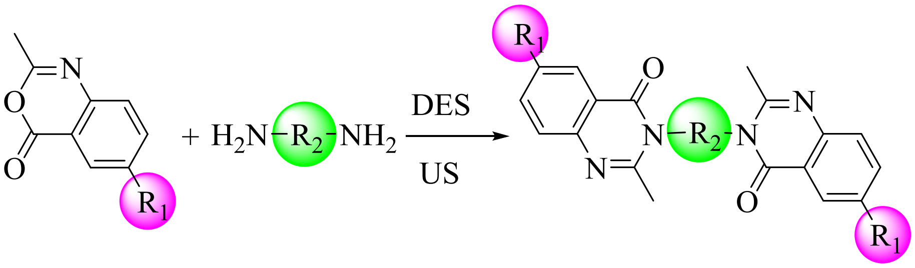

Compd.	R_1	R_2	Conc.&IC_{50}	Cells viability (%)	
				MCF-7 (µM)	A549 (µM)
**3a**	H	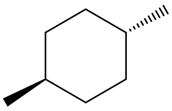	10 µM	51.00 ± 2.02	60.16 ± 1.56
			30 µM	57.95 ± 1.45	97.20 ± 0.41
			IC_{50} µM	9.78	8.15
**3b**	CH_3	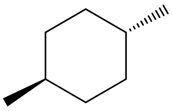	10 µM	31.52 ± 1.82	39.33 ± 2.76
			30 µM	94.90 ± 0.46	90.12 ± 0.25
			IC_{50} µM	12.70	15.70
**3c**	Cl	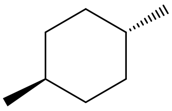	10 µM	56.64 ± 2.23	61.12 ± 1.82
			30 µM	86.85 ± 0.89	96.98 ± 0.40
			IC_{50} µM	9.05	8.14
**3d**	F	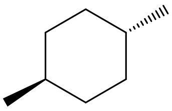	10 µM	69.73 ± 2.31	67.49 ± 0.27
			30 µM	89.10 ± 1.93	89.95 ± 043
			IC_{50} µM	7.11	8.11
**3e**	NO_2	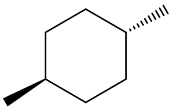	10 µM	83.12 ± 1.61	77.06 ± 1.78
			30 µM	81.18 ± 0.55	82.85 ± 1.64
			IC_{50} µM	4.55	5.34
**4a**	H	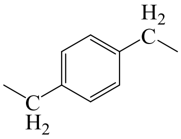	10 µM	79.06 ± 2.70	60.37 ± 3.31
			30 µM	89.85 ± 1.02	62.90 ± 1.45
			IC_{50} µM	5.38	6.11
**4b**	CH_3	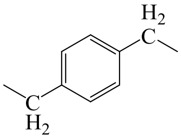	10 µM	78.02 ± 2.63	75.98 ± 2.21
			30 µM	96.50 ± 0.20	78.71 ± 1.76
			IC_{50} µM	6.73	6.79
**4c**	Cl	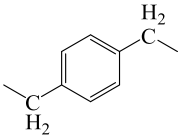	10 µM	76.84 ± 1.24	79.50 ± 2.61
			30 µM	81.08 ± 1.72	81.75 ± 0.87
			IC_{50} µM	4.65	6.07
**4d**	**F**	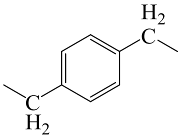	10 µM	60.25 ± 3.31	94.29 ± 0.39
			30 µM	61.92 ± 1.01	96.09 ± 0.244
			IC_{50} µM	6.19	5.55
**4e**	NO_2	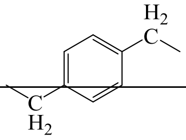	10 µM	95.58 ± 0.26	80.22 ± 2.10
			30 µM	95.26 ± 0.90	87.03 ± 1.45

			IC_{50} µM	2.75	2.98
**5a**	H	$-CH_2-CH_2-$	10 µM	51.03 ± 1.92	32.44 ± 2.91
			30 µM	58.07 ± 1.55	88.68 ± 1.83
			IC_{50} µM	9.79	12.32
**5b**	CH_3	$-CH_2-CH_2-$	10 µM	27.07 ± 1.70	39.99 ± 2.11
			30 µM	87.65 ± 1.24	89.67 ± 1.76
			IC_{50} µM	13.55	15.73
**5c**	Cl	$-CH_2-CH_2-$	10 µM	80.22 ± 2.10	61.87 ± 2.34
			30 µM	87.03 ± 1.45	82.10 ± 1.93
			IC_{50} µM	6.12	7.98
**5d**	**F**	$-CH_2-CH_2-$	10 µM	76.84 ± 1.24	54.49 ± 0.27
			30 µM	81.08 ± 1.72	89.95 ± 043
			IC_{50} µM	5.39	7.95
**5e**	NO_2	$-CH_2-CH_2-$	10 µM	91.11 ± 1.87	91.54 ± 0.56
			30 µM	92.80 ± 2.52	97.83 ± 0.24
			IC_{50} µM	5.21	5.05
**6a**	H	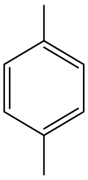	10 µM	75.50 ± 2.17	54.49 ± 0.27
			30 µM	79.02 ± 0.90	89.95 ± 043
			IC_{50} µM	6.98	8.41
**6b**	CH_3	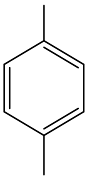	10 µM	61.13 ± 2.35	59.16 ± 1.53
			30 µM	82.84 ± 0.92	95.12 ± 0.22
			IC_{50} µM	7.48	8.13
**6c**	Cl	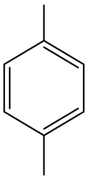	10 µM	94.78 ± 0.49	91.98 ± 0.49
			30 µM	96.88 ± 0.21	98.02 ± 0.21
			IC_{50} µM	5.53	5.15
**6d**	**F**	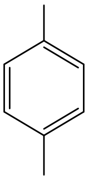	10 µM	54.22 ± 2.78	82.65 ± 1.67
			30 µM	86.56 ± 1.54	81.78 ± 0.45
			IC_{50} µM	5.70	4.54
**6e**	NO_2	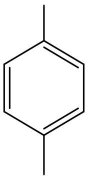	10 µM	95.26 ± 0.45	95.40 ± 0.24
			30 µM	95.87 ± 0.21	97.60 ± 0.21
			IC_{50} µM	2.73	3.43
**7a**	H	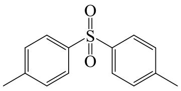	10 µM	72.68 ± 0.79	82.73 ± 1.63
			30 µM	89.08 ± 0.94	82.10 ± 0.50
			IC_{50} µM	4.24	4.58

**7b**	CH_3	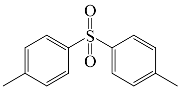	10 µM	53.03 ± 2.29	53.13 ± 2.20
			30 µM	86.90 ± 1.24	86.84 ± 1.19
			IC_{50} µM	5.78	5.77
**7c**	Cl	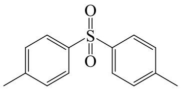	10 µM	93.82 ± 0.49	72.34 ± 0.78
			30 µM	96.21 ± 0.34	88.43 ± 0.19
			IC_{50} µM	3.91	4.21
**7d**	**F**	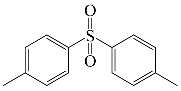	10 µM	61.03 ± 2.82	96.46 ± 0.65
			30 µM	78.09 ± 1.79	96.68 ± 0.17
			IC_{50} µM	1.46	3.45
**7e**	NO_2	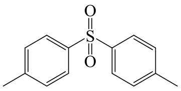	10 µM	81.08 ± 1.77	95.99 ± 0.14
			30 µM	86.97 ± 1.26	96.76 ± 0.87
			IC_{50} µM	1.26	2.75
**11**	H	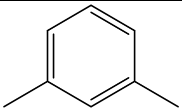	10 µM	61.80 ± 1.11	79.8 ± 1.05
			30 µM	88.54 ± 1.09	83.20 ± 1.02
			IC_{50} µM	5.42	5.94
Sorafenib			10 µM	69.85 ± 1.02	89.8 ± 1.14
			30 µM	90.79 ± 1.43	91.21 ± 1.44
			IC_{50} µM	4.03	5.20

Green Metrics Calculations[1,2]

$$\textbf{\% Atomic Efficiency (AE)} = \frac{\text{Mol Wt. of desired product}}{\text{Mol Wt. of all reagents}} \times 100$$

$$\textbf{\% Carbon Efficiency (CE)} = \frac{\text{Mass of carbon in product}}{\text{Totall mass of carbon in the reactants}} \times 100$$

$$\textbf{Reaction Mass Efficiency (RME)} = \frac{\text{Mass of the isolated product}}{\text{Total mass of reactants used in the reaction}} \times 100$$

$$\textbf{\% Yield Economy (YE)} = \frac{\text{Reaction percent}}{\text{Time in min}} \times 100$$

$$\textbf{E-Factor (EF)} = \frac{\text{Mass of the total waste}}{\text{Mass of the crude product}}$$

$$\textbf{Process Mass Intensity (PMI)} = \frac{\text{Total mass used in process}}{\text{Mass of product}}$$

References

1. D. Curzons, D. J. C. Constable, D. N. Mortimer and V. L. Cunningham, *Green Chem.*, 2001, **3**, 1-6.
2. C. Jimenez-Gonzalez, D. J. C. Constable and C. S. Ponder, *Chem. Soc. Rev.*, 2012, **41**, 1485-1498.
